# CD4 T cells control development and maintenance of brain-resident CD8 T cells during polyomavirus infection

**DOI:** 10.1371/journal.ppat.1007365

**Published:** 2018-10-29

**Authors:** Taryn E. Mockus, Shwetank, Matthew D. Lauver, Heather M. Ren, Colleen S. Netherby, Tarik Salameh, Yuka Imamura Kawasawa, Feng Yue, James R. Broach, Aron E. Lukacher

**Affiliations:** 1 Department of Microbiology and Immunology, Penn State College of Medicine, Hershey, PA, United States of America; 2 Department of Biochemistry and Molecular Biology, Penn State College of Medicine, Hershey, PA, United States of America; 3 Department of Pharmacology, Penn State College of Medicine, Hershey, PA, and; 4 Institute for Personalized Medicine, Penn State College of Medicine, Hershey, PA, United States of America; Brown University, UNITED STATES

## Abstract

Tissue-resident memory CD8 T (T_RM_) cells defend against microbial reinfections at mucosal barriers; determinants driving durable T_RM_ cell responses in non-mucosal tissues, which often harbor opportunistic persistent pathogens, are unknown. JC polyomavirus (JCPyV) is a ubiquitous constituent of the human virome. With altered immunological status, JCPyV can cause the oft-fatal brain demyelinating disease progressive multifocal leukoencephalopathy (PML). JCPyV is a human-only pathogen. Using the mouse polyomavirus (MuPyV) encephalitis model, we demonstrate that CD4 T cells regulate development of functional antiviral brain-resident CD8 T cells (bT_RM_) and renders their maintenance refractory to systemic CD8 T cell depletion. Acquired CD4 T cell deficiency, modeled by delaying systemic CD4 T cell depletion until MuPyV-specific CD8 T cells have infiltrated the brain, impacted the stability of CD8 bT_RM_, impaired their effector response to reinfection, and rendered their maintenance dependent on circulating CD8 T cells. This dependence of CD8 bT_RM_ differentiation on CD4 T cells was found to extend to encephalitis caused by vesicular stomatitis virus. Together, these findings reveal an intimate association between CD4 T cells and homeostasis of functional bT_RM_ to CNS viral infection.

## Author summary

Tissue resident memory cells (T_RM_) persist in nonlymphoid organs serving as frontline defense against microbial reinfection. The requirements for generating pathogen-specific T_RM_ to acutely resolved infections is well documented; however, little is known about the development of T_RM_ to persistent infections. In this study, we investigated the importance of CD4 T cell availability to CD8 T_RM_ development during persistent viral encephalitis. Using mouse polyomavirus (MuPyV) brain infection and systemic CD4 T cell insufficiency, we found that loss of CD4 T cells abrogated brain T_RM_ (bT_RM_) development, disrupted the metabolic homeostasis of CD8 T cells, and reduced CD8 T cell responses upon viral reinfection. Additionally, CD8 T cells in CD4 T cell-deficient mice required resupply from circulating CD8 T cells, directly contrasting the independence from circulation in canonical T_RM_. Upon delayed CD4 T cell depletion and brain infection with an acutely resolving viral infection, CD8 T cells also had aberrant bT_RM_ development and required resupply from the vasculature. Our findings demonstrate that CD4 T cells are essential for establishing long-lasting, virus-specific CD8 bT_RM_ for antiviral CNS immunosurveillance. Our data also raise important clinical implications for developing therapies to augment CD4 T cell help to bolster protective CD8 bT_RM_ responses.

## Introduction

Tissue-resident memory T cells (T_RM_), the largest memory T cell subset, are non-recirculating cells parked in both nonlymphoid and lymphoid tissues [1–3]. The importance of CD8 T_RM_ cells in limiting infections, their distinct transcriptional profile, the signals driving their differentiation, and their capacity to control reinfections at mucosal portals of pathogen entry are well documented [1, 4]. Far less is known about the requirements for establishing CD8 T_RM_ cells in non-mucosal tissues, particularly those populated by large populations of non-renewable cells, such as the brain, where rapid control of infection may prove lifesaving. Recent studies using acutely resolved viral meningo-encephalitidies have revealed the durability of brain-resident memory CD8 (bT_RM_) cells and their role in clearing CNS viral infections [5]. However, little is known of the requirements for establishing and maintaining CD8 T_RM_ cells to persistent viral CNS infections.

Polyomaviruses are natural pathogens that persist as silent, lifelong infections in healthy hosts of many vertebrates. Thirteen polyomaviruses to date have been identified as constituents of the human virome, but several [BKPyV, JCPyV, and Merkel cell polyomavirus (MCPyV)] are opportunistic pathogens known to cause life-threatening diseases in immunocompromised individuals [6]. JCPyV is acquired in early adolescence probably via gastrointestinal routes of infection, reaches seropositivity rates over 60% by sixty years of age, and persists in the kidney, urinary tract, bone marrow, and possibly the brain [7, 8]. With altered immune status as a consequence of HIV/AIDS, immune-modulating therapeutics for autoimmune diseases (e.g., natalizumab for relapsing-remitting multiple sclerosis), and biologic anti-cancer agents, JCPyV can cause progressive multifocal leukoencephalopathy (PML) [6]. Polyomaviruses productively infect and persist only in their host reservoir species. An acknowledged impediment to understanding PML pathogenesis and the immunovirologic factors that put patients at risk for PML is the absence of tractable animal models [9]. Human astrocytes/oligodendrocytes engrafted in brains of immune and myelin deficient (RAG2^-/-^MBP^shi/shi^) mice support JCPyV replication and virus-induced loss of these glial cells results in demyelination [10]. However, deciphering the immunological deficits that predispose patients to PML remains to be determined.

CD4 T cells are necessary for regulating the phenotype and function of CD8 memory T cells in lymphoid organs [11]. In acute viral infections, CD8 T cells primed in the absence of CD4 T cells (“unhelped” CD8 T cells) lose the ability to produce effector cytokines such as IFN-γ, TNF-α, IL-2, as well as the cytolytic protein granzyme B, and are unable to control primary infection or infections by reencountered pathogens [12–14]. Furthermore, memory differentiation is aberrant in unhelped CD8 T cells, as demonstrated by impaired upregulation of L-selectin (CD62L), IL-7Rα (CD127), and the CD27 costimulatory molecule [15, 16]. Recall responses of unhelped memory CD8 T cells to infection with vaccinia virus are restrained by PD-1 [17], and vaccine-elicited unhelped CD8 T cells express multiple inhibitory receptors [18]. Unhelped CD8 T cells infiltrate the brain in response to vesicular stomatitis virus (VSV) [19] and lymphocytic choriomengitis virus (LCMV) [5]. Other models of central nervous system (CNS) viral infection, however, suggest that CD4 T cell help is necessary for CD8 T cell function and CD8 bT_RM_ development. Unhelped CD8 T cells cannot control West Nile Virus (WNV) infection and gradually lose the ability to produce effector cytokines [20]. CD8 T cells in the CNS of CD4 T cell-deficient mice inoculated intracerebrally with a neurotropic mouse coronavirus had reduced IFN-γ and granzyme B expression, impaired viral control, disrupted memory differentiation, and increased apoptosis [21–23]. CD4 T cell help to CD8 T cells and B cells is also pivotal in the control of measles virus encephalitis [24, 25]. For PML, it is interesting to note a case report documenting isolation of JCPyV DNA carrying a mutation that ablates a JCPyV-specific CD4 T cell epitope [26]. These studies highlight the discrepant data on the dependence of CD4 T cell help for sustaining CD8 bT_RM_ formation during CNS viral infections, and point toward the possibility that such CD4 T cell dependence may be context-dependent.

Mouse polyomavirus (MuPyV) is a ubiquitous natural mouse pathogen that establishes a lifelong infection [6, 27]. MuPyV infects a wide variety of cells such as epithelial cells, mesenchymal cells, macrophages, and dendritic cells [28, 29]. MuPyV persistently infects multiple organs including the spleen, brain, kidney, and bone marrow, with the site of inoculation affecting the organ distribution of persistent viral infection [30]. Mice lacking secondary lymphoid organs fail to generate an anti-MuPyV CD8 T cell response [31]. Previous work has shown that CD8 T cells contribute a large part of the host defense against MuPyV infection in the periphery [32, 33].

In this study, we asked whether CD4 T cell help was essential for generating CD8 bT_RM_ in mice infected with MuPyV. Upon intracerebral (i.c.) MuPyV inoculation, virus-specific CD8 T cells are recruited to the brain and establish a CD8 bT_RM_ population [34–36]. MuPyV inoculated i.c. spreads systemically [34]. We found that unhelped virus-specific CD8 T cells infiltrated the brain and were functional during early stages of MuPyV infection, but failed to control virus during reinfection. We previously described the contrast in dependence of brain-infiltrating CD4 T cells, but not of CD8 T cells, on their circulating counterparts [34]. Here, we found that maintenance of unhelped CD8 T cells required resupply from CD8 T cells in the vasculature. The transcriptome of unhelped CD8 T cells showed disruption of genes involved in pathways of T_RM_ function and homeostasis. Moreover, CD4 T cell insufficiency impaired differentiation of functional virus-specific CD8 bT_RM_ not only at the stage of naïve CD8 T cell priming, but also after MuPyV-specific CD8 T cells had infiltrated the brain. The importance of CD4 T cells for homeostasis of virus-specific CD8 T cells during a persistent viral encephalitis has clear clinical ramifications for establishing durable immunosurveillance of persistent CNS infections.

## Results

### CD4 T cells are dispensable for the recruitment, maintenance, and function of brain-infiltrating MuPyV-specific CD8 T cells

**Fig 1:**
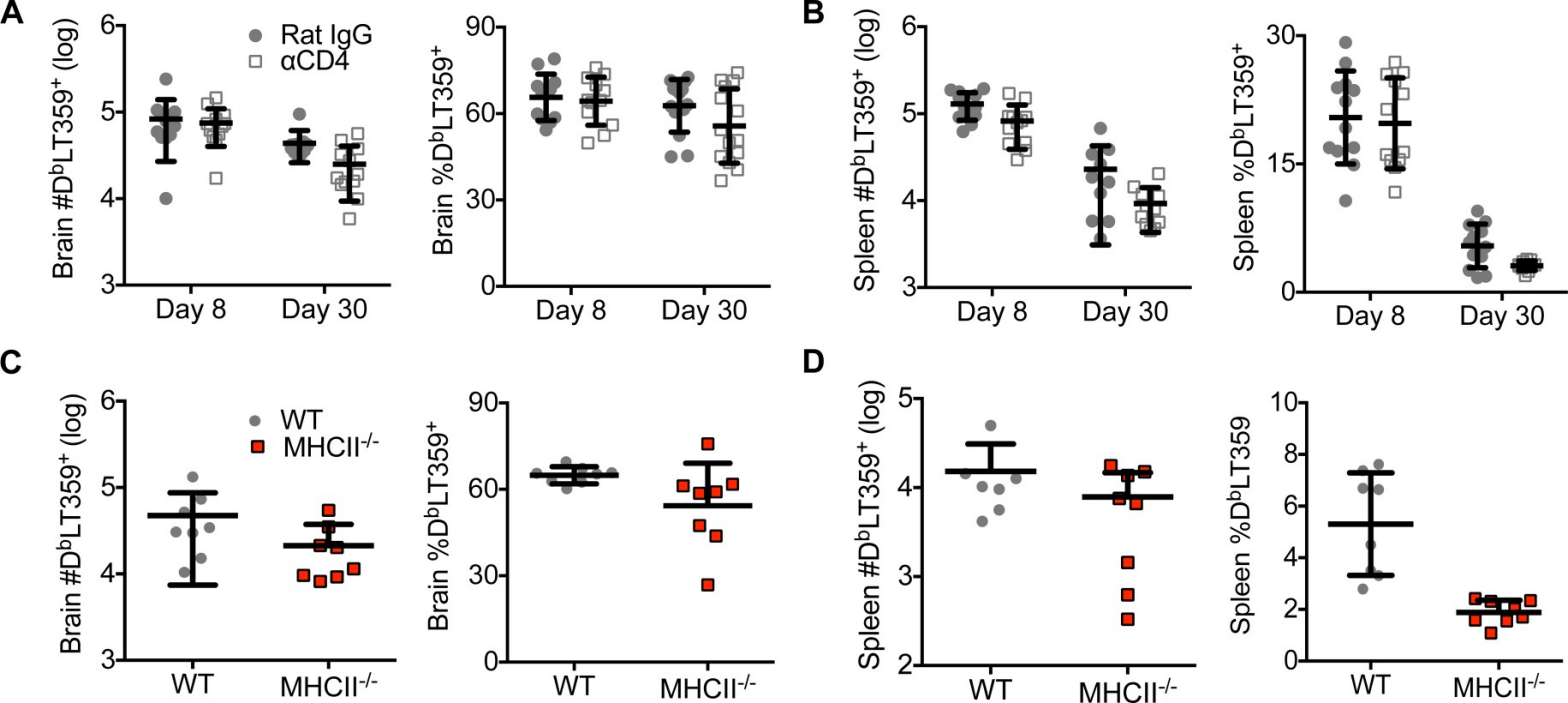
Brain and spleen MuPyV-specific CD8 T cell responses in CD4 T cell-sufficient and—deficient mice. **(A, B)** Number (left) and frequency (right) of CD44^hi^ D^b^LT359 tetramer^+^ CD8 T cells in brain (A) and spleen (B) during acute (day 8 p.i.) and persistent (day 30 p.i.) infection. **(C, D)** Number (left) and frequency (right) of CD8^+^ CD44^hi^ D^b^LT359 tetramer^+^ in brain (C) and spleen (D) during persistent (day 30 p.i.) infection in WT and MHCII^-/-^ mice. Mean ± SD of 7–10 mice per group from 2 independent experiments.

A large body of evidence has shown that CD4 T cell deficiency during recruitment of naïve CD8 T cells has negative consequences on memory CD8 T cell differentiation [11]. To ask whether availability of CD4 T cell help during priming of virus-specific CD8 T cells affected recruitment and maintenance of CD8 T cells during MuPyV encephalitis, CD4 T cells were depleted by intraperitoneal (i.p.) administration of CD4 mAb before MuPyV infection and weekly thereafter until endpoint. CD4 T cell-sufficient and -deficient mice showed similar frequency and number of CD8 T cells specific for the dominant D^b^LT359 epitope in both the brain and spleen in acutely [day 8 post-infection (p.i.)] and persistently (day 30 p.i.) infected mice (**Fig 1A & 1B)**. The helped and unhelped virus-specific CD8 T cell responses in the spleen decreased similarly between days 8 and 30 p.i. (**Fig 1B).** In contrast, the frequency and number of virus-specific CD8 T cells in the brain did not significantly change between days 8 and 30 p.i. in either CD4 T cell-sufficient or–deficient mice **(Fig 1A**). In addition, unhelped MuPyV-specific CD8 T cells in the brain proliferated similarly as compared to helped CD8 T cells and expressed Bcl-2 (**S1A & S1B Fig**). Independent confirmation of these results was made using MHC II^-/-^ mice inoculated i.c. with MuPyV, where no differences were found in the frequency or number of virus-specific CD8 T cells in the brains of MHC II^-/-^ and wild type (WT) mice (**Fig 1C & 1D**). This equivalence in helped vs unhelped virus-specific CD8 T cell responses in the brain mirrors that reported for WT and MHC II^-/-^ mice given VSV i.n. [19]. CD4 T cell availability did not affect the pattern of effector/memory differentiation of MuPyV-specific CD8 T cells in either the brain or spleen based on surface co-expression of KLRG1 and CD127, and expression of the transcription factors T-bet, eomesodermin (eomes), TCF-1, and Blimp-1 (**S1C–S1H Fig**).

**Fig 2:**
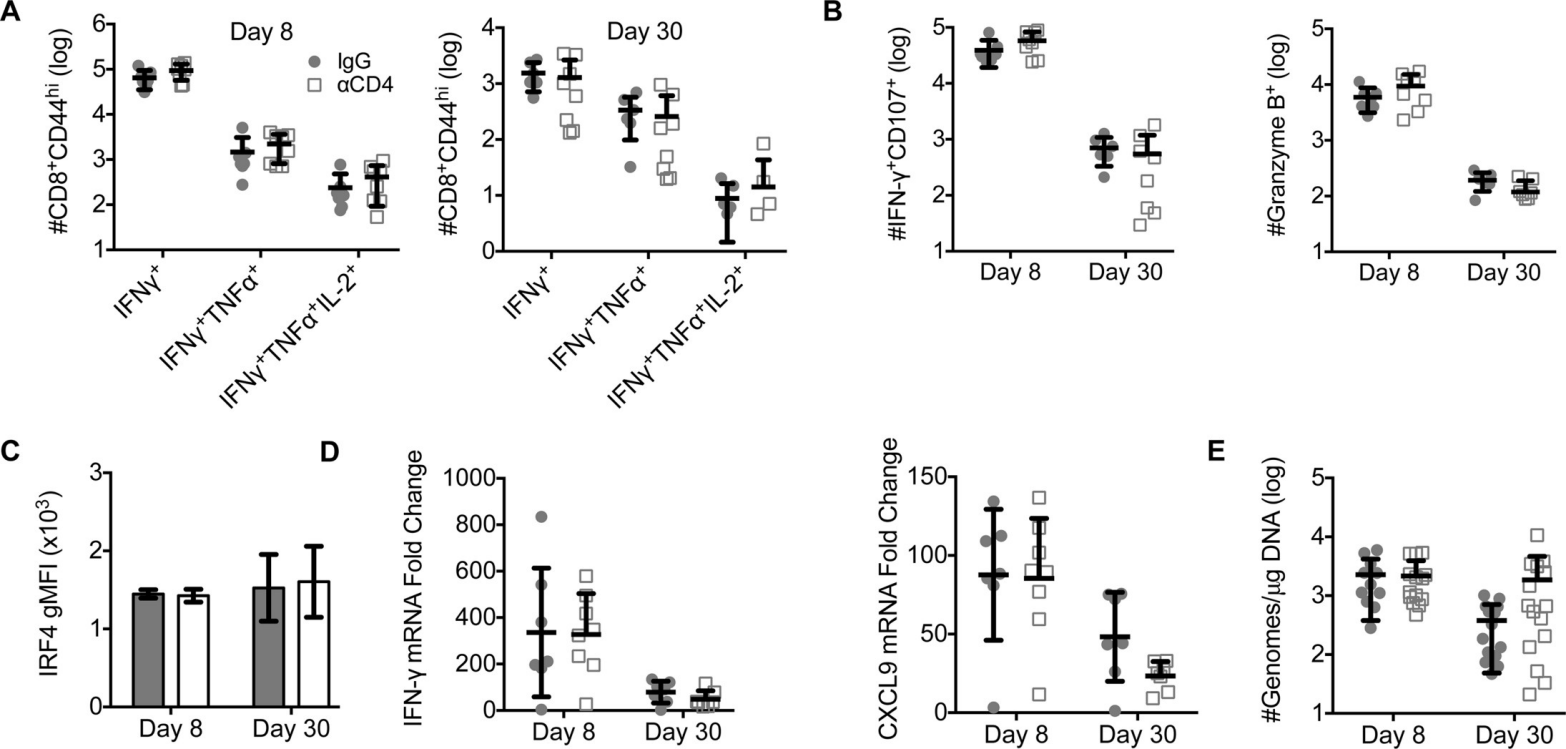
Unhelped virus-specific CD8 T cells in the brain remain functional. **(A)** Number of IFN-γ^+^, IFN-γ ^+^ TNF-α^+^, and IFN-γ ^+^ TNF-α^+^ IL-2^+^ CD44^hi^ CD8 T cells from brains at days 8 (left) and 30 (right) p.i. following ex vivo stimulation with LT359 peptide. **(B)** Number of IFN-γ^+^ CD107^+^ (left) and Granzyme B^+^ CD44^hi^ (right) CD8 T cells from brain at days 8 and 30 p.i. There was a significant decrease in the number of IFN-γ^+^ CD107^+^ (P<0.0001) and Granzyme B^+^ (P<0.0001) between days 8 and 30 p.i. **(C)** Ex vivo staining for IRF4 in helped and unhelped D^b^LT359 tetramer^+^ CD8 T cells isolated from brain. **(D)** Quantitative PCR analysis for fold change of IFN-γ (left) and CXCL9 (right) mRNA compared to housekeeping gene 18s rRNA at days 8 and 30 p.i. There was a significant decrease in IFN-γ (P = 0.0002) and CXCL9 (P = 0.0003) mRNA between days 8 and 30 p.i. **(E)** Real-Time PCR analysis of viral genome copies from brains at days 8 and 30 p.i. Mean ± SD of 7–10 mice per group from 2 independent experiments (A-D) or 13–15 mice per group from 3 independent experiments (E), two-way ANOVA with Tukey’s multiple comparisons test (A-E).

We next asked whether unhelped CD8 T cells exhibited functional deficits. Previous studies have shown that CD4 T cell help is necessary for the development of functionally competent CD8 T cells [11]. In contrast, similar numbers of helped and unhelped D^b^LT359-specific CD8 T cells produced IFN-γ, TNF-α, and IL-2, and retained cytotoxic effector potential (i.e., intracellular granzyme B and peptide-induced CD107 cell surface expression) during acute and persistent MuPyV infection (**Fig 2A & 2B)**. In the spleen, however, fewer unhelped D^b^LT359-specific CD8 T cells produced IFN-γ in persistently infected mice (**S2A Fig**). Helped and unhelped D^b^LT359-specific CD8 T cells in the brain had similar sensitivity to antigen stimulation, as evidenced by the expression of IRF4 (**Fig 2C**), a transcription factor upregulated by TCR engagement [37]. Furthermore, IFN-γ mRNA and CXCL9 mRNA, an IFN-γ-induced chemokine, were upregulated compared to uninfected control mice similarly in brains of CD4 T cell-sufficient and -deficient mice (**Fig 2D)**. Although no difference in viral load was observed in brains of CD4 T cell-deficient and -sufficient mice during acute infection, viral loads trended higher during persistent infection in the absence of CD4 T cells (**Fig 2E**). Thus, CD4 T cells appear not to overtly impact the magnitude, differentiation, or function of virus-specific CD8 T cells infiltrating the brains of MuPyV-infected mice.

### CD4 T cells are essential for the development of bT_RM_

**Fig 3:**
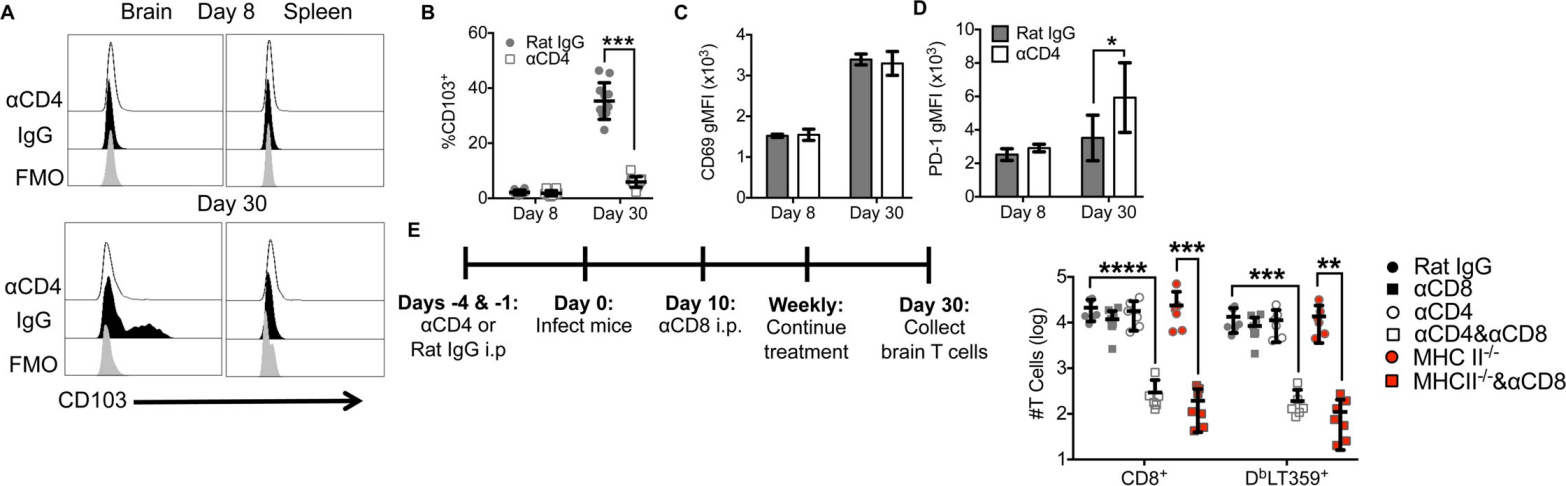
Unhelped MuPyV-specific CD8 T cells in the brain are not maintained upon systemic CD8 T cell depletion. **(A)** Representative histogram of CD103^+^ and CD103^-^ D^b^LT359 tetramer^+^ CD8 T cells from brain and spleen at days 8 and 30 p.i. **(B)** Frequency of CD103^+^ D^b^LT359 tetramer^+^ CD8 T cells from brain at days 8 and 30 p.i. **(C, D)** CD69 (C) and PD-1 (D) gMFI on D^b^LT359 tetramer^+^ CD8 T cells from brain at days 8 and 30 p.i. **(E)** Experimental design, and number of total CD8 T cells and D^b^LT359 tetramer^+^ CD8 T cells from brains at day 30 p.i. Mean ± SD of 8–12 mice per group from 3 independent experiments (A-D) or 6–10 mice per group from 3 independent experiments (E). *P<0.05, **P<0.01, ***P<0.001, ****P<0.0001, two-way ANOVA with Tukey multiple comparisons test.

As we recently reported, approximately 40% of D^b^LT359-tetramer^+^ CD8 T cells in the brain express CD103 in persistently infected mice [35]. In CD4 T cell-deficient mice, few CD103^+^ MuPyV-specific CD8 T cells were detected in the brain 30 days after MuPyV inoculation (**Fig 3A & 3B** and **S3A Fig**), although these cells expressed CD69 at levels similar to those in CD4 T cell-sufficient mice (**Fig 3C)**. During WNV infection of the brain, TGF-β produced from regulatory T cells is important for the upregulation of CD103 [38]. In our model, FoxP3^+^CD25^+^ CD4 T cells infiltrate the brain but constitute only 5% of CD44^+^ CD4 T cells in WT mice (**S3B Fig**). After stimulation with PMA/ionomycin, brain CD4 T cells showed a transient 4-fold increase in TGF-β mRNA compared to unstimulated CD4 T cells (**S3C Fig**). IL-21 has also been associated with establishing CD8 T_RM_ and their expression of CD103 [39]. CD4 T cells produced >100-fold more IL-21 mRNA after PMA/ionomycin stimulation (**S3D Fig**). Together, these data support the possibility that TGF-β and IL-21 contribute to upregulating CD103 on the virus-specific CD8 T cells during MuPyV infection. Furthermore, unhelped virus-specific CD8 T cells had higher PD-1 expression compared to helped virus-specific CD8 T cells (**Fig 3D** and **S3E & S3F Fig**). Diminished expression of CD103, a commonly used marker of T_RM_ cell differentiation, and elevated PD-1 expression raised the possibility that CD4 T cell help qualitatively modulated MuPyV-specific CD8 bT_RM_ residing in the brain.

We recently demonstrated that systemic depletion of CD8 T cells after their entry into the brain did not impact their maintenance, while i.p. administration of a depleting CD4 mAb led to a dramatic decline in numbers of CD4 T cells in the brain [34]. These data indicated that brain-resident CD8 and CD4 T cells during MuPyV encephalitis showed a dichotomy in their dependence on cells in the circulation. We asked whether maintenance of unhelped MuPyV-specific CD8 T cells in MuPyV-infected mouse brain retained independence from the vascular compartment. To do this, CD8 T cell-depleting mAb was given at day 10 p.i., which was after MuPyV-specific CD8 T cells had infiltrated the brain [34] (**Fig 3E**). In CD4 T cell-sufficient mice, depletion of circulating CD8 T cells had no effect on the number of total CD8 T cells or D^b^LT359-specific CD8 T cells in the brain at day 30 p.i. **(Fig 3E**). In marked contrast, the number of total CD8 T cells and D^b^LT359-specific cells declined approximately 100-fold in CD4 T cell-deficient mice depleted of circulating CD8 T cells at this timepoint (**Fig 3E)**. Together, these data suggest that CD4 T cell availability for development and maintenance of CD8 bT_RM_ is critical during persistent viral CNS infections.

**Fig 4:**
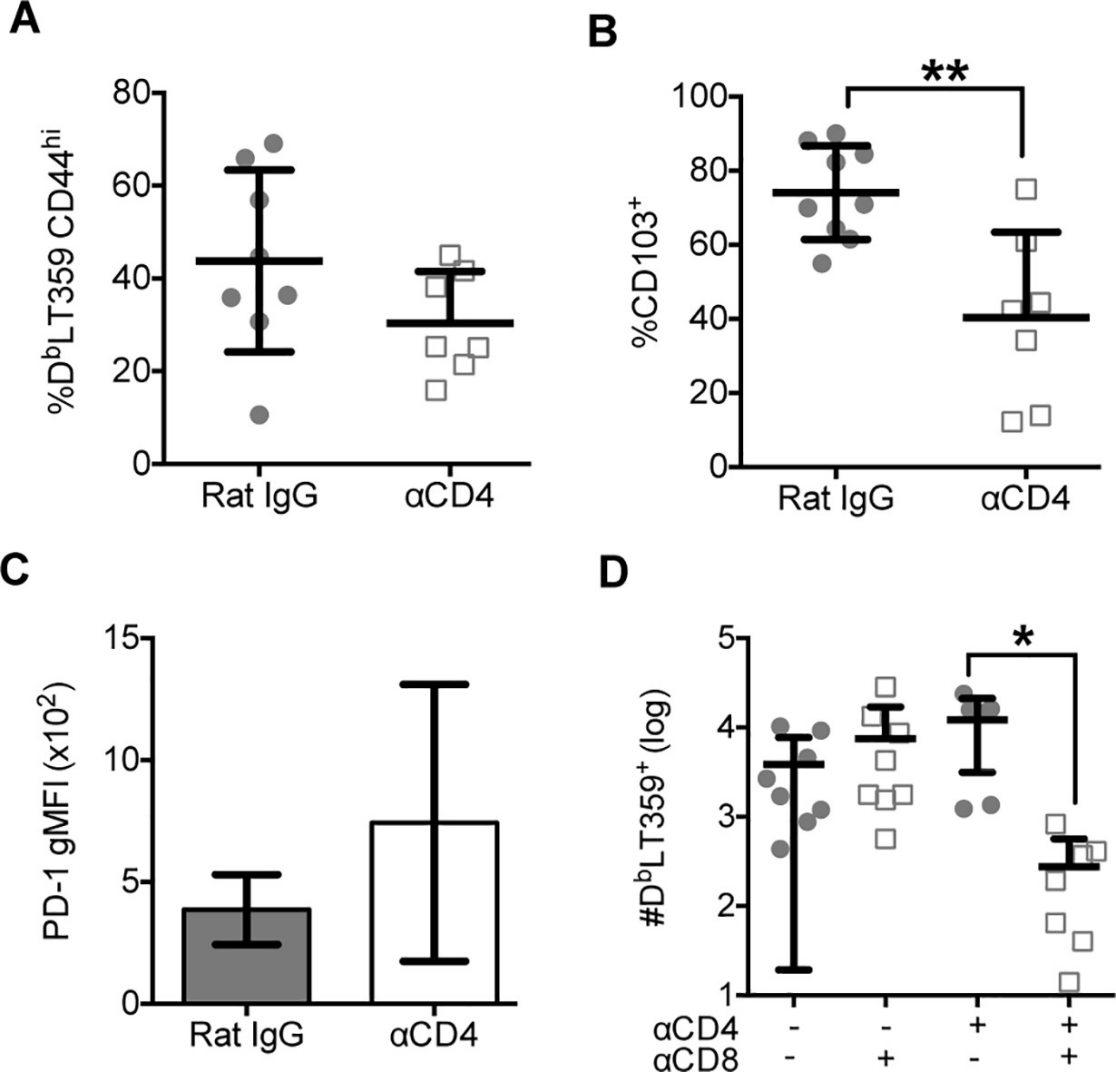
CD4 T cell help is essential for the development of bT_RM_ in response to VSV infection. **(A)** Frequency of D^b^LT359 tetramer^+^ CD8 T cells from brains 30 days post-VSV infection. **(B)** Frequency of CD103^+^ D^b^LT359 tetramer^+^ CD8 T cells from brains 30 days post-VSV infection. **(C)** gMFI of PD-1 expression on D^b^LT359 tetramer^+^ CD8 T cells. **(D)** Number of D^b^LT359 tetramer^+^ CD8 T cells from brains 30 days post-VSV infection. Mean ± SD of 3–4 mice per group from 2 independent experiments. *P<0.05, **P<0.01, Mann-Whitney test (A-C) and one-way ANOVA (D).

A differential dependence of CD4 T cell help on development of virus-specific CD8 T_RM_ in different viral systems may depend on the type of viral infection. To explore this possibility, we used a recombinant vesicular stomatitis virus encoding the D^b^LT359 epitope (rVSV-LT359) [40]. CD4 T cell-sufficient and -deficient mice had similar frequencies of D^b^LT359-specific CD8 T cells in the brain 30 days after rVSV-LT359 intranasal (i.n.) inoculation (**Fig 4A**). This result confirms that of Wakim et al. who found no differences in antigen-specific CD8 T cell responses between WT and CD4 T cell-deficient mice in brains of mice with VSV encephalitis [19]. We further observed that unhelped virus-specific CD8 T cells in brains after i.n. rVSV-LT359 inoculation failed to upregulate CD103 (**Fig 4B**). PD-1 expression on unhelped CD8 T cells, however, was not significantly higher (**Fig 4C**). Systemic CD8 T cell depletion resulted in loss of D^b^LT359-specific CD8 T cells in CD4 T cell-depleted mice, but not in CD4 T cell-sufficient mice given αCD8 (**Fig 4D**). Using primers against VSV genomic RNA (gRNA), we were able to detect low levels of VSV gRNA during persistence in both CD4 T cell-sufficient and–deficient mice (**S4 Fig**). Similarly, a previous study reported persistent VSV gRNA after i.n. infection, but detected no VSV mRNA at the same time point [41]. Collectively, these data indicate that CD4 T cell help is essential for generating CD8 bT_RM_ in both VSV and MuPyV CNS infections.

To exclude the possibility that antibody-mediated CD4 T cell depletion increased the permeability of the blood brain barrier (BBB) and allowed CNS access by anti-CD8α, we measured extravasation of sodium fluorescein into brains of CD4 T cell-sufficient and -deficient mice 10 days after MuPyV infection. CD4 T cell-deficient mice showed no change in the concentration of sodium fluorescein dye in the brain, indicating that the integrity of the BBB was unaltered by systemic CD4 T cell depletion (**S5A Fig**). Furthermore, systemically administered CD8 T cell-depleting mAb did not stain CD8 T cells in the brain parenchyma, irrespective of CD4 T cell status (**S5B Fig**). Unhelped virus-specific CD8 T cells expressed the adhesion molecules VLA-4, PSGL1, and LFA-1, suggesting that CD4 T cell availability did not alter the ability of these cells to home to and traffic into the infected brain (**S5C Fig**). To ask whether unhelped CD8 T cells remained in the vasculature and, thus, directly exposed to depleting anti-CD8α, we performed intravascular staining with FITC-conjugated CD45 mAb. No difference in the ratio of extravascular to intravascular total and virus-specific CD8 T cells was seen between helped and unhelped mice (**S5D Fig**). Collectively, these data confirm that bT_RM_ become dependent on hematogenous replenishment in the absence of CD4 T cell help.

### CD4 T cells promote MuPyV-specific CD8 bT_RM_ function

**Fig 5:**
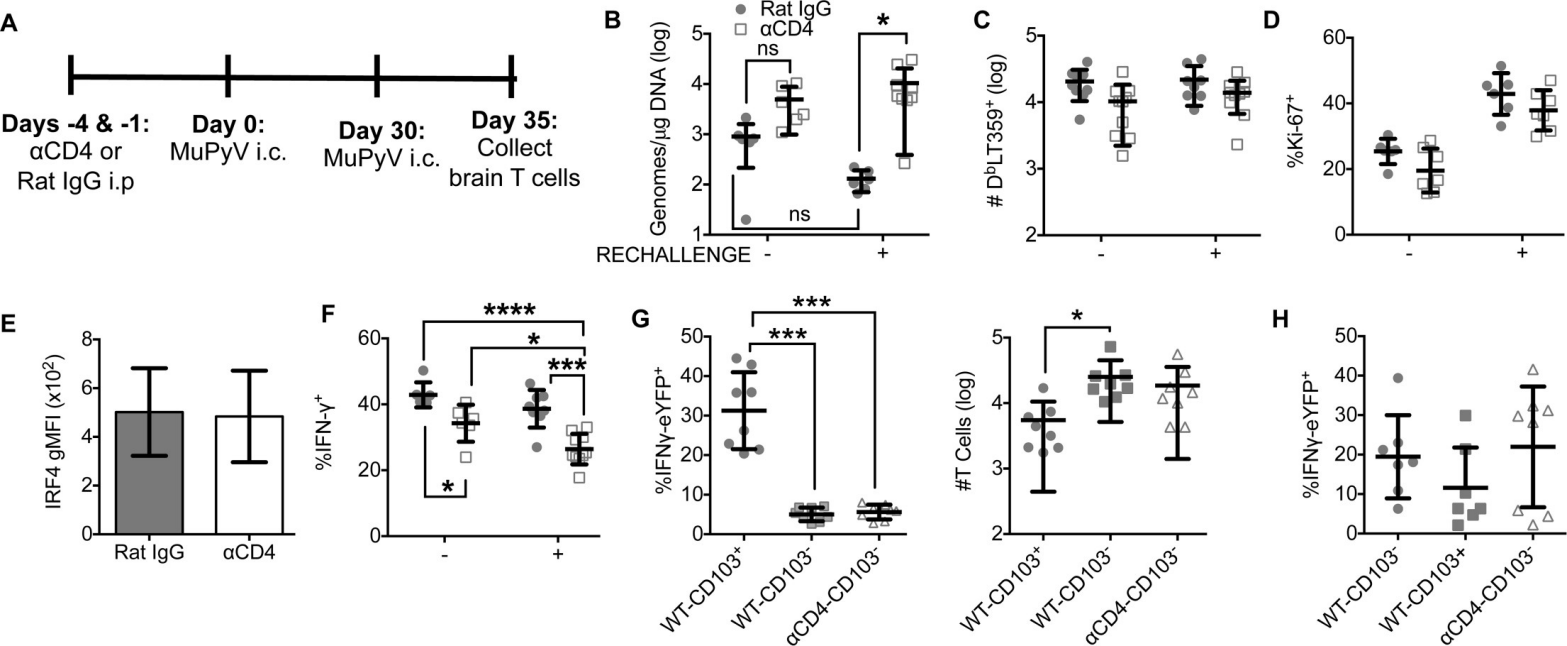
Reduced effector activity of unhelped CD8 T cells in brain upon reinfection. **(A)** Experimental design. **(B)** Real-Time PCR analysis of viral genome copies from brain. **(C)** Number of D^b^LT359 tetramer^+^ CD8 T cells from brain. **(D)** Frequency of Ki-67^+^ D^b^LT359 tetramer^+^ cells from the brain. **(E)** Ex vivo staining for IRF4 in D^b^LT359 tetramer^+^ CD8 T cells. **(F)** Frequency of IFN-γ^+^ CD44^hi^ CD8 T cells from brain following ex vivo stimulation with LT359 peptide. **(G)** Frequency (left) of IFNγ-eYFP^+^ cells and number (right) of D^b^LT359 tetramer^+^ WT-CD103^+^,WT-CD103^-^ cells, and αCD4-CD103^-^ cells from the brains of IFNγ-eYFP mice at day 5 post reinfection. **(H)** Frequency of IFNγ-eYFP^+^ cells 30 days p.i. Mean ± SD of 9–10 mice per group from 3 independent experiments (A-F) or 8 mice per group from two independent experiments (G, H). ns = not significant, *P<0.05, ***P<0.001, Two-Way ANOVA with Tukey multiple comparisons test (B-F) and one-way ANOVA (G,H).

A central defect of unhelped memory CD8 T cells in lymphoid tissues is their failure to expand upon reencountering cognate antigen [42]. T_RM_ accelerate control of viral reinfection in nonlymphoid tissues [1]; however, a requirement for CD4 T cell help for bT_RM_ to retain their recall response capability is unknown. We previously showed that MuPyV-infected mice, which possess potent neutralizing virus antibodies, mount recall responses in the brain after i.c. challenge with homologous MuPyV [36]. We asked whether availability of CD4 T cell help affects recall responses of virus-specific CD8 T cells to MuPyV reinfection and ability to control the challenge infection. Mice were depleted of circulating CD4 T cells before i.c. inoculation with MuPyV and then reinfected i.c. with MuPyV at day 30 p.i. (**Fig 5A)**. At day 5 after reinfection, viral load was significantly higher in CD4 T cell-deficient mice reinfected with MuPyV compared to CD4 T cell-sufficient mice with reinfection (**Fig 5B**). However, the viral load was not significantly higher than CD4 T cell-deficient mice receiving mock rechallenge (**Fig 5B**). Although the viral load was trending lower in rechallenged CD4 T cell-sufficient mice, the difference did not reach statistical significance (**Fig 5B**). This loss of viral control was observed despite similar numbers and proliferation of brain virus-specific CD8 T cells **(Fig 5C & 5D**). Although MuPyV-specific CD8 T cells proliferated rapidly in the reinfected mice, no significant increase was seen in the number of D^b^LT359^+^ CD8 T cells in the brain upon rechallenge. This discrepancy between cell proliferation and numbers suggests engagement of a concurrent cell death process. Interestingly, no difference in IRF4 was seen between CD4 T cell helped and unhelped virus-specific CD8 T cells, implying comparable levels of TCR activation (**Fig 5E**). Yet, the frequency of IFN-γ^+^ CD8 T cells upon *ex vivo* LT359 peptide stimulation was lower in rechallenged CD4 T cell-deficient than -sufficient mice (**Fig 5F)**; although significant, there was <10% difference between the mock vehicle injected persistently infected rat IgG-treated and CD4 T cell-deficient groups. Using IFN-γ eYFP reporter mice to visualize effector function by MuPyV-specific CD8 T cells *in situ*, we found that a significantly higher fraction of CD103^+^ than CD103^-^ cells produced IFN-γ upon reinfection, with CD103^-^ D^b^LT359 tetramer^+^ CD8 T cells in both CD4 T cell-sufficient and -deficient mice producing little IFN-γ (**Fig 5G**). This defect was not due to a decrease in CD103^-^ cells in brain (**Fig 5G**). Without rechallenge, CD103^-^ T cells from CD4 T cell -sufficient and -deficient mice have similar IFNγ-eYFP production to CD103^+^ CD8 T cells (**Fig 5H**). These results indicate that CD4 T cells during recruitment and maintenance of CD8 bT_RM_ are necessary for effective control of MuPyV CNS reinfection and improved ability to produce IFN-γ.

**Fig 6:**
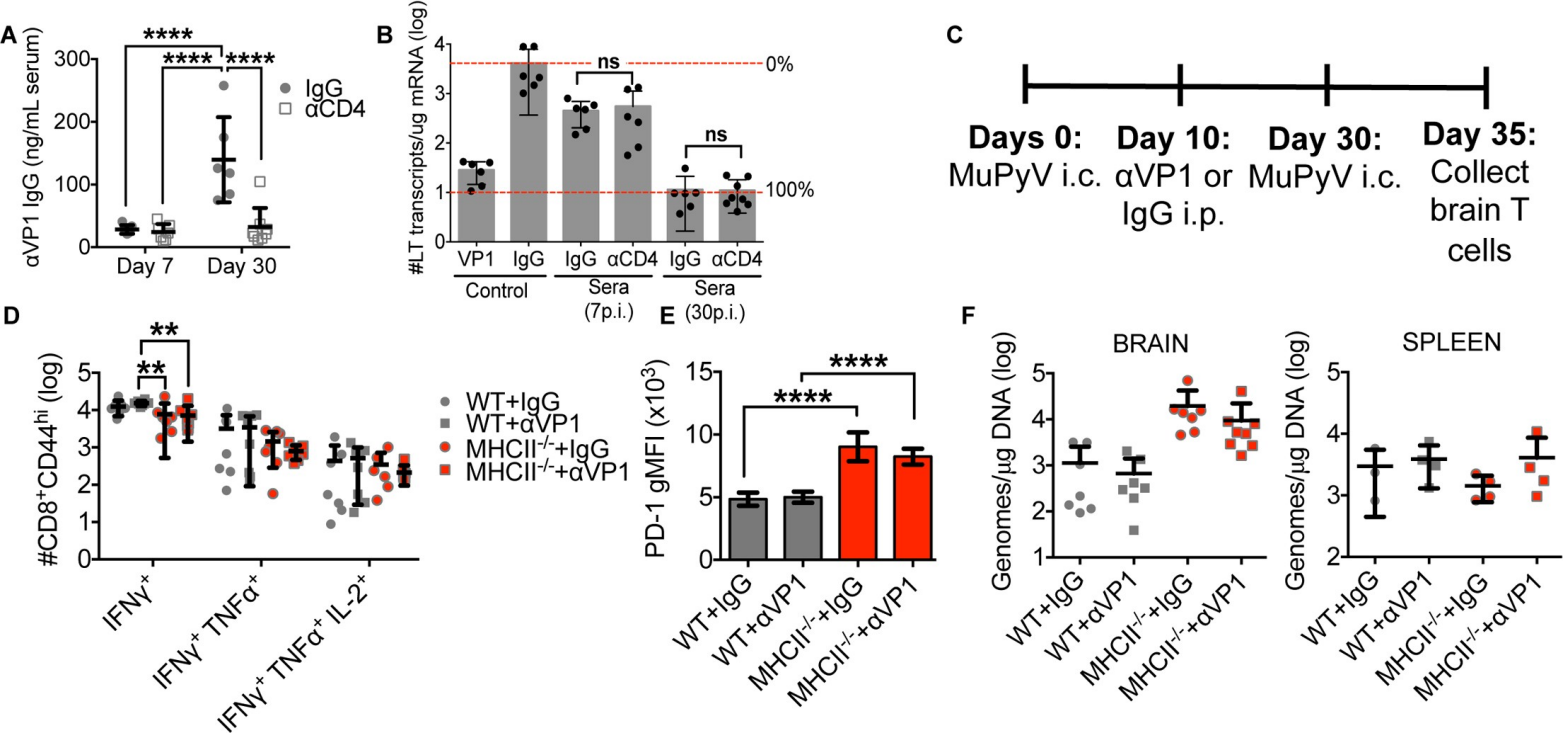
Unhelped CD8 T cell functionality upon reinfection is independent of neutralizing antibodies. **(A)** Anti-VP1 IgG levels in serum at days 7 and 30 p.i. **(B)** Neutralization assay of antibodies from serum at days 7 and 30 p.i. Assay controls indicate cells treated with only IgG or VP1 mAb. **(C)** Experimental design. **(D)** Frequency of IFN-γ^+^, IFN-γ ^+^ TNF-α^+^, and IFN-γ ^+^ TNF-α^+^ IL-2^+^ CD44^hi^ CD8 T cells from brains 5 days post rechallenge stimulated ex vivo with LT359 peptide. **(E)** PD-1 gMFI on D^b^LT359^+^ CD8 T cells. **(F)** Real-Time PCR analysis of viral genome copies from brain five days post rechallenge. Mean ± SD of 7–8 mice per group from two independent experiments (A-E) or 4 mice per group from one experiment (F). ns = not significant, *P<0.05, ****P<0.001, two-way ANOVA with Tukey multiple comparisons test (D) and one-way ANOVA (E).

Absence of virus-neutralizing antibodies may be associated with increased viral burden, with the consequent high antigen levels driving virus-specific T cell dysfunction. However, the contribution of antiviral antibodies to offsetting T cell exhaustion depends on the experimental viral system. MuPyV infection elicits a virus-neutralizing CD4 T cell-independent IgG response directed to VP1, the major polyomavirus capsid protein [43, 44]. Similarly, influenza virus infection also generates a T cell-independent influenza-specific IgG that helps resolve primary influenza infection and prevents reinfection [45]. Despite a decrease in αVP1 IgG titers in CD4 T cell-depleted mice at day 30 p.i., sera from CD4 T cell-deficient and -sufficient mice exhibited strong virus-neutralization capability during acute and persistent infection (**Fig 6A & 6B**). To formally exclude an effect of MuPyV-neutralizing antibodies on peripheral viral load and T cell function during MuPyV rechallenge, WT and MHC II^-/-^ mice were passively immunized with a neutralizing VP1 IgG mAb [46] from day 10 p.i. to MuPyV i.c. reinfection at day 30 p.i (**Fig 6C**). Despite passive immunization, unhelped virus-specific CD8 T cells still exhibited significant deficits in IFN-γ production (**Fig 6D**), while PD-1 expression was increased compared to CD4 T cell-sufficient mice (**Fig 6E**). Viral loads were similar in MHCII^-/-^ mice with and without mAb VP1 treatment (**Fig 6F**). Serum from MHCII^-/-^ and WT mice that were passively immunized with αVP1 possessed similar virus-neutralization capabilities (**S6A Fig**). These data demonstrate that virus-specific antibodies did not rescue the unhelped CD8 T cell response.

### Helped and unhelped MuPyV-specific CD8 T cells in the brain have distinct transcriptomes

**Fig 7:**
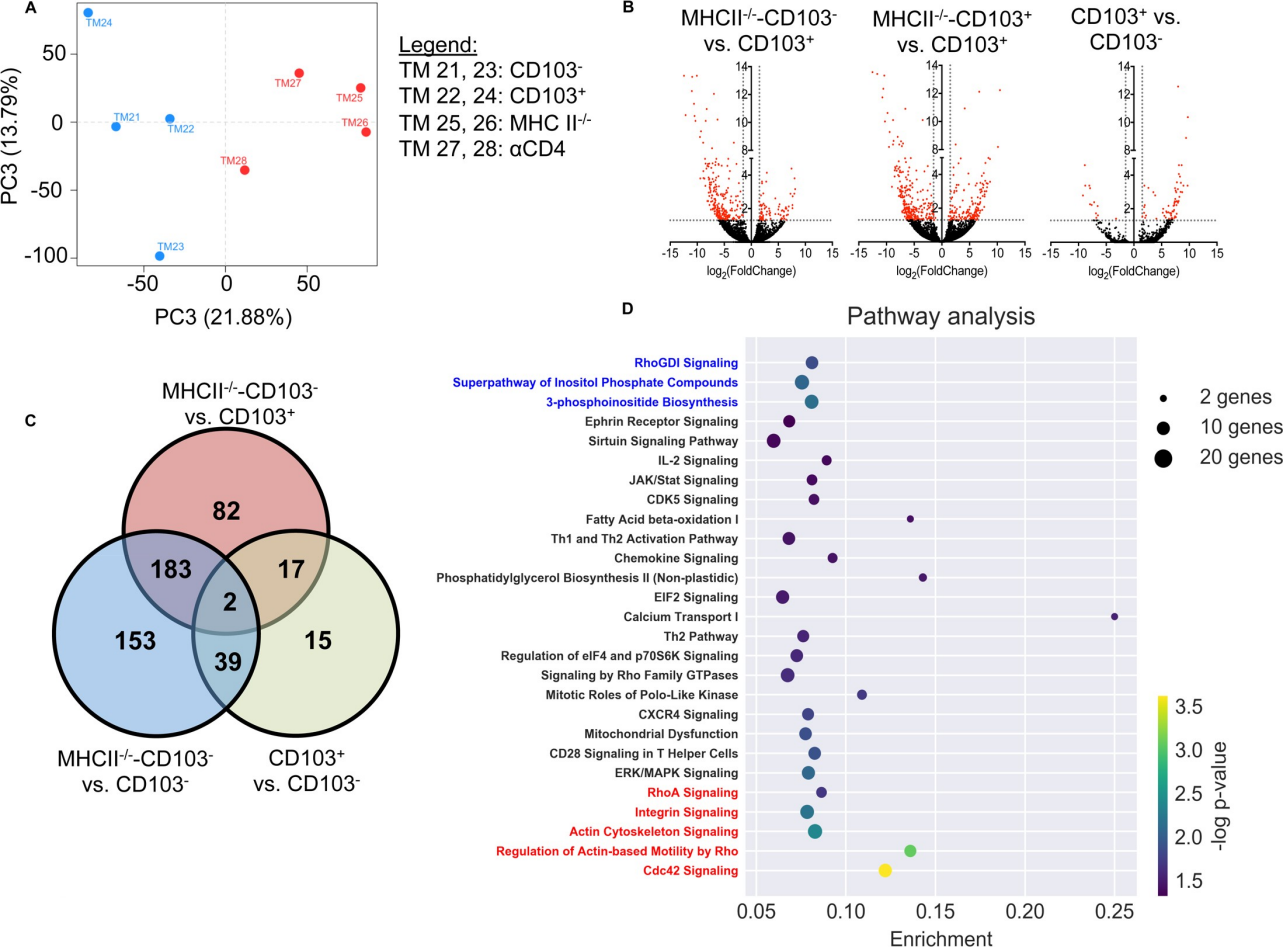
Helped and unhelped MuPyV-specific CD8 T cells in the brain have distinct transcriptional profiles. **(A)** Principal component analysis of FACS-sorted D^b^LT359 tetramer^+^ CD8 T cells pooled from brains of 3–4 MuPyV (each pool designated as TM) i.c. inoculated mice at days 30–40 p.i. Red dots, CD4 T cell-insufficient mice; blue dots, WT mice. **(B)** Volcano plot representation of differential expression analysis of transcripts of MHCII^-/-^ vs WT-CD103^+^ (left) MHCII^-/-^ vs CD103^-^ (middle), and CD103^+^ vs CD103^-^ (right). Red represents differentially expressed transcripts over the cut-off of a q-value ≤ 0.05 (y-axis represents -log10[q-value]) and the X-axis represents the fold change. **(C)** Venn diagram showing the number of differentially expressed genes in MHCII^-/-^-CD103^-^ vs CD103^+^ (top), MHCII^-/-^-CD103^-^ vs CD103^-^ (left bottom), and CD103^+^ vs CD103^-^ (right bottom). **(D)** Ingenuity pathway analysis of differentially expressed transcripts between MHCII^-/-^-CD103^-^ and CD103^-^. Y-axis represents pathway and X-axis represents enrichment factor. Bubble size represents the number of differentially expressed transcripts and color represents the P-value calculated by Fisher’s Exact test. Blue labels indicate upregulated pathways and red labels indicate downregulated pathways with a cut-off z score ≤ 2.0.

The common defect in IFN-γ production by CD103^-^ MuPyV-specific CD8 T cells in WT and CD4 T cell-deficient mice led us to survey the transcriptional landscape of CD103^+^ and CD103^-^ D^b^LT359 tetramer^+^ CD8 T cells sorted from brains of persistently infected WT and MHCII^-/-^ mice (**S7A Fig**); for this analysis, we refer to D^b^LT359 tetramer^+^ CD8 T cells from MHC-II^-/-^ mice as MHCII^-/-^-CD103^-^ CD8 T cells and D^b^LT359 tetramer^+^ CD103^-^ and CD103^+^ from WT mice as CD103^-^ and CD103^+^. 377 transcripts were differentially expressed between CD103^-^ and MHCII^-/-^-CD103^-^ CD8 T cells, whereas 267 transcripts were differentially expressed between CD103^+^ and MHCII^-/-^-CD103^-^ CD8 T cells (**Fig 7A–7C**). Only 73 transcripts, however, were differentially expressed between helped CD103^-^ and CD103^+^ CD8 T cells (**Fig 7A–7C**). These data reveal that CD103^-^ and CD103^+^ cells had similar transcriptomes, both of which were substantially different from the transcriptomes of MHC II^-/-^-CD103^-^ cells. Our recent report showing similar phenotype and function by brain-resident, MuPyV-specific CD8 T cells irrespective of CD103 expression [35] are in line with the highly overlapping transcriptomes of CD103^+^ and CD103^-^ CD8 T cells. These findings support accumulating evidence that caution is warranted when considering CD103 as a stereotypical marker of T_RM_ differentiation [1, 36]. Ingenuity pathway analysis of MHCII^-/-^-CD103^-^ vs CD103^-^ CD8 T cells revealed significant aberrations in the activation state and homeostasis of unhelped CD8 T cells. MHCII^-/-^-CD103^-^ exhibited significant downregulation of pathways including cdc42, actin cytoskeleton remodeling, and actin-based motility, which are essential for cell migration (**Fig 7D**). Additionally, MHCII^-/-^-CD103^-^ CD8 T cells had downregulated RhoA signaling, which has recently been identified as a central regulator of CD4 T cell viability, proliferation, and migratory capacity in the CNS of EAE mice [47]. Notably, MHCII^-/-^-CD103^-^ CD8 T cells had significant downregulation of Runx3 (**S1 Table**), a recently identified component of the transcription factor signature of CD8 T_RM_ [48]. MHCII^-/-^-CD103^-^ CD8 T cells also showed significant upregulation of phosphoinositide pathways **(Fig 7D**). Gain-of-function mutations in phosphoinositide pathways have been reported to promote exhaustion and senescence of CD8 T cells [49, 50]. MHCII^-/-^-CD103^-^ CD8 T cells differentially expressed genes involved in mitochondrial function; mitochondrial dysfunction is highly prevalent in CD8 T cells isolated from HIV^+^ patients [51]. By comparison to MuPyV-specific CD8 T cells in brains of MHC-II^-/-^ mice, the CD103^+^ and CD103^-^ cells in brains of WT mice shared most of the same gene expression pathways (**S7B Fig)**. Thus, unhelped CD8 T cells during persistent MuPyV infection have a profoundly altered transcriptome in a pattern indicating defective homeostasis and activation.

### CD4 T cells are necessary for MuPyV-specific CD8 bT_RM_ homeostasis

**Fig 8:**
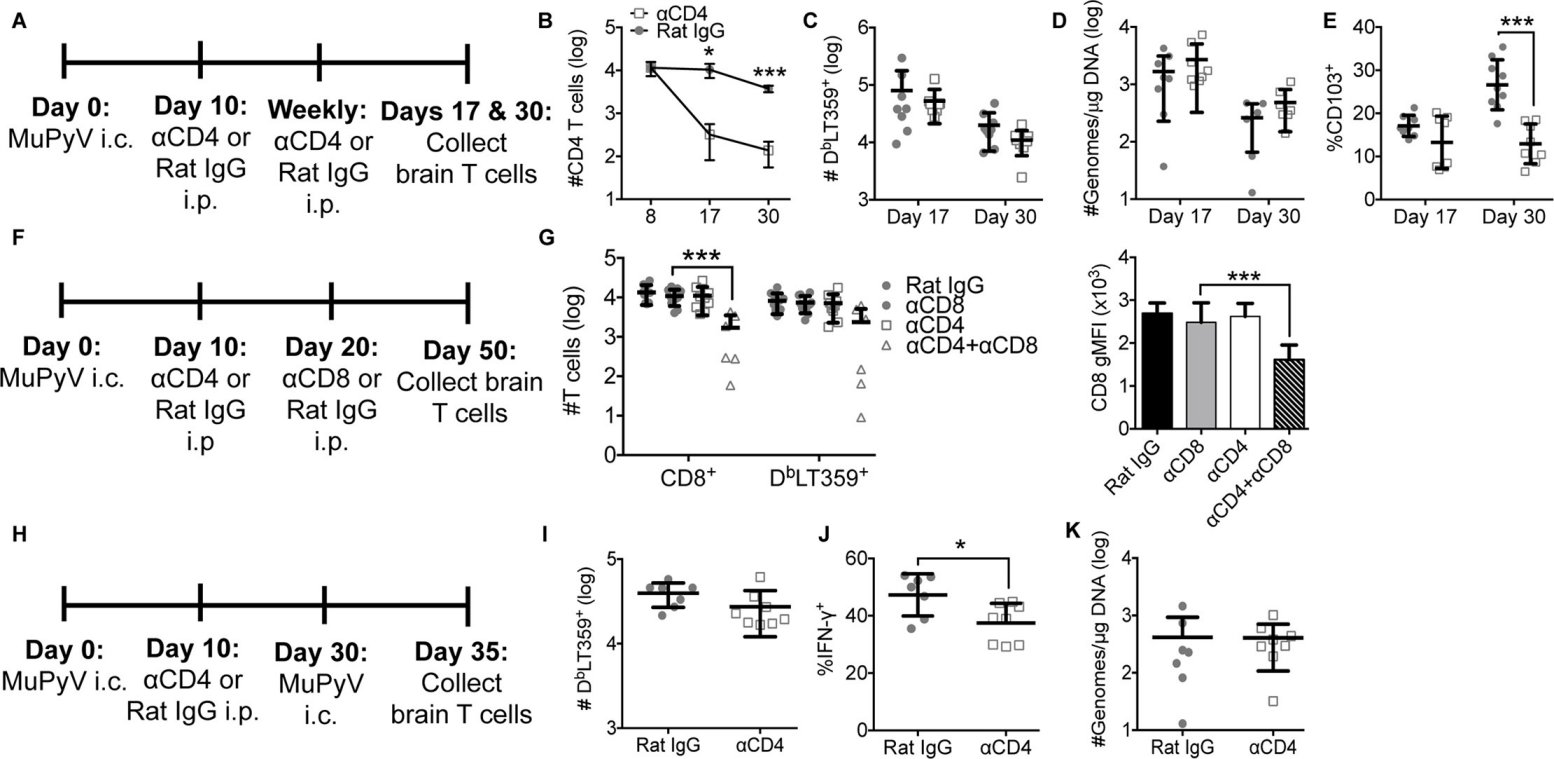
Delayed systemic CD4 T cell depletion affects bT_RM_ differentiation/maintenance. **(A)** Experimental design. **(B)** Number of CD4 T cells in brain. **(C)** Number of virus-specific CD8 T cells from brain at 17 and 30 days p.i. **(D)** Real-Time PCR analysis of viral genome copies from brain. **(E)** Frequency of CD103^+^ D^b^LT359-specific CD8 T cells in brain at days 17 and 30 p.i. There was a significant increase in CD103^+^ CD8 T cells (p = 0.0132) in Rat IgG-treated mice between days 8 and 30 p.i. **(F)** Experimental design. **(G)** Number of total and virus-specific CD8 T cells (left) and gMFI of CD8 on D^b^LT359 tetramer^+^ CD8 T cells (right) in brain upon systemic depletion of CD4 and/or CD8 T cells. **(H)** Experimental design. **(I)** Number of D^b^LT359 tetramer^+^ CD8 T cells from brain. **(J)** Frequency of IFNγ^+^ CD44^hi^ CD8 T cells from brain upon ex vivo stimulation with LT359 peptide. **(K)** Real-Time PCR analysis of viral genome copies from brain. Mean ± SD of 8 mice per group from two independent experiments. *P<0.05, ***P<0.001, two-way ANOVA with Sidak’s multiple comparisons test (B-E,G), one-way ANOVA (G), and Mann-Whitney test (I-K).

Because CD4 T cell deficiency is often an acquired rather than an inherited condition, we asked whether delayed systemic deletion of CD4 T cells affected development of functionally competent CD8 bT_RM_ during MuPyV encephalitis. We therefore started i.p. administration of CD4 T cell-depleting mAb at day 10 p.i. (**Fig 8A**). The number of CD4 T cells significantly declined in the brain with systemic anti-CD4 depletion (**Fig 8B**). Although no difference was seen in the number of virus-specific CD8 T cells or viral load in the brain with delayed CD4 T cell depletion (**Fig 8C & 8D**), the frequency of virus-specific CD103^+^ CD8 T cells was significantly lower at 30 days p.i. compared to CD4 T cell-sufficient mice (**Fig 8E**). Because the frequency of CD103^+^ D^b^LT359-specific CD8 T cells was significantly reduced in CD4 T cell-deficient mice, we asked whether a decline in CD4 T cells affected development of virus-specific CD8 T_RM_ cells after CNS infiltration. To this end, systemic CD4 T cell depletion began at day 10 p.i., coupled with circulating CD8 T cells at day 20 p.i. (**Fig 8F**). The number of total CD8 T cells and MuPyV-specific CD8 T cell and the gMFI of CD8 on the MuPyV-specific CD8 T cells were significantly reduced in CD4 T cell-deficient mice (**Fig 8G**). We have previously published that increased CD8 gMFI marks bT_RM_ [34]. We next assessed the ability of these unhelped D^b^LT359-specific CD8 T cells to control MuPyV challenge infection (**Fig 8H**). Five days after reinfection, the number of D^b^LT359-specific CD8 T cells was not significantly different between CD4 T cell-sufficient and -deficient mice (**Fig 8I**), but the frequency of IFN-γ-producing cells was significantly lower (**Fig 8J**). Virus levels, however, were the same between CD4 T cell-sufficient and–deficient mice (**Fig 8K**). These data support the concept that CD4 T cells are required to sustain functional CD8 bT_RM_ to persistent viral encephalitis.

## Discussion

CD4 T cells modulate the differentiation program of pathogen-specific CD8 T cells that establish permanent residence as memory cells in mucosal barrier tissues, but their role in driving T_RM_ development in non-barrier tissues is less understood [11]. In this study, we determined that CD4 T cell help was essential for establishment and maintenance of CD8 bT_RM_ to MuPyV encephalitis. CD4 T cells guided the differentiation of MuPyV-specific CD8 bT_RM_ during naïve T cell priming and were required for maintenance of functional antiviral CD8 bT_RM_ in brains of persistently infected mice. Notably, CD4 T cell insufficiency resulted in diminished effector competence of antiviral CD8 T cells encountering MuPyV reinfection in the brain. An ongoing dependence on CD4 T cells for induction and maintenance of virus-specific CD8 bT_RM_ to a persistent CNS infection has clear clinical implications for individuals whose immune status is altered by infection or immunomodulatory therapeutic agents.

Accumulating evidence supports the likelihood that JCPyV adapts to selective pressure applied by virus-specific CD4 T cells. JCPyV recovered from PML patients carry mutations in the VP1 capsid protein that affect binding to sialylated glycans, which serve as receptors for JCPyV entry into host cells [52]. Thus, JCPyV-PML VP1 mutations are thought to alter viral tropism and endow JCPyV with neuropathic potential. Recent evidence, however, supports the alternative possibility that these VP1 mutations serve an immune evasion purpose by preventing recognition by neutralizing antibodies [53] and by ablating VP1-specific CD4 T cell epitopes [26]. These findings have motivated efforts to isolate monoclonal antibodies capable of broadly cross-neutralizing wild type and VP1 mutant JCPyVs to protect patients at-risk of and as therapeutics for PML [54]. In the pre-combination antiretroviral therapy era, PML had an approximately 5% incidence in patients with HIV/AIDS, a disease initiated by profound CD4 T cell deficiency [55]. Individuals with idiopathic CD4 T cell lymphopenia, which manifests without overt changes in CD8 T cells, B cells, or NK cells, are also at elevated risk for PML [56]. JCPyV VP1/LT-specific CD8 T cell adoptive immunotherapy in a PML patient drove viral DNA below PCR detectability in the CSF and improved neurological status [57]. Our data together with these studies suggest that preserving and augmenting anti-JCPyV CD4 T cells or providing “helper” cytokines in PML-susceptible individuals, and using such interventions to supplement CD8 T cell immunotherapy for PML, could promote differentiation of brain-infiltrating CD8 T cells into CD8 bT_RM_ and improve disease prognosis.

Upon LCMV infection in the periphery, CD4 T cell deficiency is associated with sustained high viral load, which in turn, upregulates checkpoint inhibitory receptors (e.g., PD-1) on virus-specific CD8 T cells. Blockade of these receptors drives recovery of effector competence and control of persistent infection [58–60]. In the MuPyV system, however, CD4 T cell deficiency did not result in higher virus levels, and helped and unhelped CD8 T cells had equivalent functional competence (**Fig 2**). Yet, PD-1 expression was increased on unhelped virus-specific CD8 T cells (**Fig 2 and S3 Fig**). Similarly, unhelped CD8 T cells that infiltrate HSV-1-infected sensory ganglia upregulate PD-1 and retain effector functionality [61]. Additionally, HSV-1 latency is maintained [61]. Together, these data raise the intriguing possibility that the major role of CD8 T cells in the persistently infected CNS may be to control resurgent viral infection.

RNA-seq analyses revealed profound differences in the transcriptomes of helped vs unhelped MuPyV-specific CD8 T cells from the brains of persistently infected mice. Pathway analyses point toward significant defects in cell migration by unhelped CD8 T cells, which may impair their ability to co-localize with virus-infected cells. Changes in mitochondrial function and loss of RhoA signaling pathways in unhelped CD8 T cells suggest that unhelped CD8 T cells may inadequately survey the infected tissue due to their defective T cell activation and metabolism [47, 62]. Also, depressed functional integrity of unhelped CD8 T cells may necessitate resupply of new effector T cells from the circulation for their maintenance in the brain.

The nature of CD4 T cell help changes over the course of persistent viral infections. A large body of literature documents that the magnitude of the CD8 T cell response during persistent viral infections is regulated, in part, by IL-21 and IL-2 produced by CD4 T cells [63–65]. Other studies, however, have shown that IL-10, usually considered an immunosuppressive cytokine, can promote maturation of memory CD8 T cells [66]. CD4 T cells may indirectly affect the quantity and quality of CD8 T cell responses via helping anti-viral antibody production and affinity maturation to control extent of persistent infection [11]. Maintenance of CD8 T cells during persistent MuPyV infection may also depend on de novo priming of naïve virus-specific CD8 T cells. Ongoing de novo recruitment over an infection that changes dynamically, including progressing from systemic to tissue-localized infection, could contribute to CD8 T cell heterogeneity [67]. De novo recruitment of MuPyV-specific CD8 T cells is CD4 T cell-dependent; thus, the level of availability of CD4 T cell help may conceivably regulate this avenue for CD8 T cell differentiation, including those that populate a T_RM_ cell compartment [68]. Similarly, in mice persistently infected with the neurotropic strain of mouse hepatitis virus, naïve CD4 and CD8 T cells were primed and recruited to the CNS [69]. In sum, these studies demonstrate that the nature of CD4 T cell help is dynamic.

Virus-specific CD8 T cells control infections in the CNS via cytopathic and non-cytopathic effector mechanisms [70]. We previously reported that MuPyV infection was controlled in mice lacking TNF receptors or perforin and/or Fas as efficiently as in WT mice, and that IFN-γ and IFN-I inhibited MuPyV replication in vivo [32, 33, 71]. Likewise, IFN-γ and IFN-I inhibited JCPyV replication in established human glial cell lines and primary human glial cells [72, 73]. Noteworthy is an incidental observation in a phase III clinical study to evaluate the effectiveness of IFN-γ on reducing incidence of opportunistic infections in HIV/AIDS subjects where none of the subjects in the IFN-γ cohort developed PML as opposed to a 10% incidence in the placebo group [74]. In this study, we found that reinfection with MuPyV was more efficiently controlled in CD4 T cell-sufficient than -deficient mice when CD4 T cells were systemically depleted at the time of naïve virus-specific CD8 T cell priming; however, in MuPyV-infected mice where CD4 T cell depletion was delayed, no difference in viral control was seen on i.c. reinfection (**Fig 8**). Because MuPyV-specific CD8 T cells suffered a deficit in IFN-γ functionality in early and delayed CD4 T cell-deficient situations, IFN-γ may not be the sole anti-MuPyV effector mechanism. In this connection, PML has also been diagnosed in patients with autoimmune rheumatic diseases, albeit not as frequently as in HIV/AIDS patients [75], and anti-TNFα treatment was associated with a low incidence of PML in these patients [76].

Unlike the high frequency of CD103^+^ memory CD8 T cells detected in tissues following resolution of acute viral infections [19, 77], fewer than half of MuPyV-specific CD8 T cells express CD103, an αE integrin that pairs with β7 to bind E-cadherin and retains T cells in tissues [35]. The role of CD103 in maintaining T_RM_ appears to vary between tissues; its expression is particularly important for retention in small intestine mucosal epithelium and skin epidermis [78, 79]. Interestingly, we found that virus-specific CD8 bT_RM_ expressing CD103 had superior IFN-γ activity upon CNS re-infection with MuPyV (**Fig 5**). *Yersinia pseudotuberculosis*-specific CD8 T_RM_ lacking CD103 localize with infectious foci in the intestinal lamina propria, where early inflammatory cues from infiltrating macrophages control the size of the CD103^-^ population [80]. However, no geographic differences based on CD103 expression have been described for CD8 bT_RM_ responding to brain infections [5, 81]. Studies are ongoing to define anti-MuPyV effector mechanism(s) in the CNS and potential preferential expression of effector activities by antiviral CD8 T cells.

We previously reported and confirmed here that stable maintenance of brain-infiltrating CD4 T cells to MuPyV encephalitis depends on ongoing replenishment from the vascular compartment; in sharp contrast, numbers of virus-specific CD8 T cells in the brain are unaltered by systemic CD8 T cell depletion [34]. Using the VSV encephalitis mouse model, we determined that CD4 T cell help also rendered virus-specific CD8 T cells susceptible to systemic CD8 T cell depletion. Thus, we found that the link between CD4 T cell help and establishment of CD8 bT_RM_ applies to both MuPyV and VSV encephalitis. Loss of CD4 T cells either during naïve CD8 T cell priming or after CD8 T cell effectors have accessed the CNS renders brain-localized CD8 T cells dependent on those in the circulation. Evidence presented here supports the concept that an intact systemic CD4 T cell compartment is essential for preserving a steady-state détente between CD8 bT_RM_ and persistent viral infection in the CNS.

## Materials and methods

### Ethics statement

All experiments involving mice were conducted with the approval of Institutional Animal Care and Use Committee (Protocol 46194) of The Pennsylvania State University College of Medicine in accordance with the Guide for the Care and Use of Laboratory Animals of the National Institutes of Health. The Pennsylvania State University College of Medicine Animal Resource Program is accredited by the Association for Assessment and Accreditation of Laboratory Animal Care International (AAALAC). The Pennsylvania State University College of Medicine has an Animal Welfare Assurance on file with the National Institutes of Health's Office of Laboratory Animal Welfare; the Assurance Number is A3045-01.

### Mice

Adult (6–12 wks of age) female and male C57BL/6 (B6) mice were purchased from the National Cancer Institute (Frederick, MD). Adult female and male B6.129-H2-*Ab1*^*tm1Gru*^ N12 mice (MHC class II-deficient) were purchased from Taconic Farms (Germantown, NY). Adult female and male C.129S4(B6)-*Ifng*^*tm3*.*1Lky*^*/*J mice (IFN-γ eYFP reporter) were purchased from The Jackson Laboratory (Bar Harbor, ME). Mice were bred and housed in accordance with the guidelines of the NIH Guide for the Care and Use of Laboratory Animals and the Institutional Animal Care and Use Committee at the Penn State College of Medicine.

### Viruses and infections

MuPyV.A2 was prepared in baby mouse kidney cells as described [82]. Mice were infected intracerebrally (i.c.) with 3x10^5^ PFU MuPyV.A2 in 30 μL as described [34]. For rechallenge, mice were inoculated i.c. with 3x10^5^ PFU MuPyV.A2 in 30 μl at day 0, re-inoculated i.c. with 3x10^5^ PFU MuPyV.A2 in 30 μl or vehicle at day 30 p.i., then euthanized 4–5 days later. Recombinant VSV expressing the MuPyV D^b^LT359 epitope (VSV.LT359) was grown and titered on BHK-21 cells (CCL-10; ATCC, Manassas VA) [40]. Mice were infected intranasally with 5x10^4^ PFU rVSV-LT359 diluted in PBS.

### T cell depletion and αVP1 administration

Mice were injected i.p. with 250 μg rat anti-CD4 or 250 μg rat anti-CD8α (clone GK1.5 or clone YTS169.4, respectively; Bio X Cell, West Lebanon, NH) or ChromoPure whole rat IgG (Jackson ImmunoResearch Laboratories, West Grove, PA) as indicated. Depletion was confirmed in peripheral blood by flow cytometry-based cell number assay using Absolute Count Standard (Bangs Laboratories, Fishers, IN). For passive immunization studies, the mice were injected i.p. with 250 μg rat VP1 mAb or Chromopure whole rat IgG beginning at day 10 p.i. and continuing weekly.

### T cell isolation and flow cytometry

Mononuclear cells from brains were isolated from transcardially perfused or intravascularly stained mice by collagenase-DNAse digestion and percoll gradient centrifugation as described [34]. Mononuclear cells were isolated from spleen as described [36]. For intravascular staining, animals were injected i.v. with FITC-conjugated anti-CD45 (clone 30-F11, BD Biosciences) through the tail vein three minutes before the brains were excised as described [83]. After isolation from perfused or intravascularly stained mice, cells were stained with Fixable Viability Dye (eBioscience, San Diego, CA), APC-D^b^LT359 tetramers (NIH Tetramer Core Facility, Atlanta, GA), and the following surface antibodies: CD8α (clone 53–6.7, eBioscience), CD44 (clone IM7, eBioscience), PD-1 (clone RMPI-30, Biolegend), Tim-3 (clone RMT3-23, Biolegend), 2B4 (clone m2B4(B6)4581, Biolegend), CD103 (clone M290, BD Horizon), CD69 (clone HI.2F3, Biolegend), CD49d (clone MRF4.8, Biolegend), CD162 (clone 2PH1, BD Biosciences), CD11a (clone 2D7, BD Biosciences), CD127 (clone A7R34, Biolegend), KLRG1 (clone 2F1, BD Biosciences), CD25 (clone PC61.5.3, Invitrogen), CD44 (clone IM7, BD Biosciences) and CD4 (clone RM4-5, BD Biosciences). For intracellular staining, cells were permeabilized and fixed in FoxP3 buffer fixation and permeabilization solutions (Thermo Fisher Scientific, Waltham, MA), and stained for T-bet (clone 4B10, Biolegend), eomes (clone Dan11mag, Invitrogen), Ki-67 (clone 2F1, BD Biosciences), Blimp-1 (clone 5E7, BD Biosciences), Tcf-1 (clone C6309, Cell Signaling Technologies), Granzyme B (clone GB11, BD Biosciences), IRF4 (clone IRF4.3E4, Biolegend), and Bcl-2 (clone BCL/10C4, Biolegend). For intracellular cytokine stimulation assays, lymphocytes were isolated from brain and spleen, cultured in DMEM/10% FBS for 5 h at 37°C with or without 1 μM LT359 peptide [84], stained with Fixable Viability Dye, anti-CD8α, and anti-CD44, then permeabilized and fixed in FoxP3 buffer fixation and permeabilization solutions. Intracellular staining included anti-IFN-γ (clone XMG1.2; Biolegend), anti-TNF-α (clone XMG1.2; Biolegend), anti- IL-2 (clone JES6-5H4, Biolegend), anti- CD107a (clone 1D4B, BD Biosciences), and anti-CD107b (clone ABL-93, BD Biosciences). CD4 T cells were stained with anti-CD4 and anti-CD44, permeabilized as described, and then stained with FoxP3 (clone FJK-16s, Invitrogen). Lymphocytes isolated from IFN-γ eYFP reporter mice were surface stained with anti-CD8α and anti-CD44. The intracellular signal of YFP was amplified by staining with anti-GFP (clone FM264G, Biolegend). Samples were acquired on a BD LSRFortessa (BD Biosciences, San Jose, CA) or BD LSR II (BD Biosciences) and analyzed using FlowJo software (FlowJo, LLC, Ashland, OR).

### Anti-VP1 ELISA and antibody neutralization

MuPyV major capsid protein VP1-specific ELISA were performed as described [84]. 10-fold serial dilutions of VP1 mAb [46] was used to obtain a standard curve on each of the 96-well plates, and the VP1-specific IgG concentrations were calculated using this standard. Antibody neutralization assays were conducted in NMuMG cells (CRL-1636; ATCC). 10 μg of VP1 mAb (positive control), rat IgG (negative control), or sera diluted 1:10 from MuPyV i.c. infected mice was incubated at 37°C for 1 hr with 5 x 10^3^ PFU/mL of MuPyV. The mixtures were then placed on 5 x 10^4^ adherent NMuMG cells in 12-well plates and incubated at 37°C for 1 hr. mRNA was harvested 24 hrs later and subjected to viral large T antigen quantification as previously described [85]. 0% neutralization was determined based on the mean number of LT transcripts observed with MuPyV when incubated with IgG. 100% neutralization is set at the limit of detection for the PCR assay.

### Quantitative PCR

For quantifying viral genome DNA copies, Real-Time PCR was performed on samples containing 10 ng DNA purified from brain and spleen using the Maxwell 16 nucleic acid isolation system (Promega, Madison, WI) as described [86]. For quantifying mRNA transcripts, total RNA was isolated from brain tissue per the manufacturer’s instructions. cDNA was prepared using random primers and RevertAid H Minus Reverse Transcriptase Enzyme (ThermoFisher Scientific). SYBR green quantitative PCR with gene-specific primers from IDT Technologies (Coralville, IA) for IFN-γ, TGF-β, IL-21 (fwd 5’-CTATGTGTTCTAGGAGAGATGCTG-3’, rev 5’-GGAGGAAAGAAACAGAAGCACA-3’), and CXCL9 mRNAs and 18s rRNA was performed on ABI StepOnePlus Real-Time PCR System (ThermoFisher Scientific) using previously published primer sequences [87–90]. Relative fold change over uninfected control mice was determined using the threshold cycle (2^−ΔΔ C^_T_) method [89]. For detection of VSV gRNA, total brain RNA was isolated using the Maxwell 16 simplyRNA Tissue kit. cDNA was prepared as above and SYBR green qPCR was carried out with primers amplifying VSV gRNA (fwd 5’-ATGTCACTGCAAGGCCTAAGA-3’, rev 5’-ATCTCTCCTACCGCCTGATCC). VSV genomes per copy of 18S RNA was determined by 2^(18S CT–VSV CT)^.

For the stimulation of CD4 T cells, WT mice were inoculated i.c. with MuPyV. At sacrifice, the brains were digested as described. CD4 T cells were purified from total brain homogenates using the EasyStep Mouse CD4 Positive Selection Kit II CD4 positive selection kit (Stemcell Technologies, Vancouver, Canada). Purified CD4 T cells were stimulated with PMA (50 ng/ml) and Ionomycin (1 ug/ml) for 3 hrs at 37°C. After stimulation, the cells were lysed in 1 ml of Trizol and cDNA was prepared as described above.

### Blood brain barrier (BBB) permeability assay

100 μl of 100 mg/ml sodium fluorescein dye (Sigma Aldrich, St. Louis, MO) was injected i.p. into mice at day 10 after i.c. inoculation with MuPyV.A2 or at 24 h after LPS administration (100 μg/μl). After 45 min, mice were cheekbled and transcardially perfused with PBS + 10% heparin. A 3 mm section was taken from the cerebrum, then processed as described [91] with fluorescein concentrations calculated using a standard curve.

### Immunofluorescence microscopy

Mice were treated i.p. with 250 μg of anti-CD4 or Rat IgG 4 days and 1 day before i.c. inoculation with MuPyV. At day 10 p.i., the mice received 250 μg of anti-CD8α i.p. The mice were sacrificed 15 hrs after receiving CD8 T cell depleting antibody. At sacrifice, the mice were perfused with 10mL of 10% heparin in PBS followed by 10 mL of 4% paraformaldehyde (PFA). Spleens and brains were postfixed in 4% PFA for 6 hrs and then sucrose dehydrated in 30% sucrose. 12 μm sections of brain and spleen were taken on a Leica biosystem cryostat (model CM1850, Buffalo Grove, IL). Sections were stained with rabbit anti-CD8A (Sino Biological Inc., Wayne, PA) primary antibody and goat anti-rabbit (Jackson ImmunoResearch, West Grove, PA) and goat anti-rat (Jackson ImmunoResearch) secondary antibodies.

### RNA-sequencing and gene expression pathway analysis

Mononuclear cells were isolated from brains as previously described [34] and pooled from 3–4 mice into groups designated CD103^+^, CD103^-^, and MHCII^-/-^-CD103^-^. Live cells were stained with DAPI (Sigma Aldrich, Germany), CD44, CD8, APC-D^b^LT359 tetramer, and CD103 and then sorted under BSL-2 conditions on a BD FACS Aria SORP (BD Biosciences) instrument. The collected cells were lysed with 1% IGEPAL CA-630 (Sigma-Aldrich) and immediately frozen on dry ice for storage at -80°C until further processing. The cDNA libraries were prepared using the SMARTer Ultra Low Input RNA Kit for Sequencing–v4 (TAKARA Bio, CA) and Nextera XT DNA Library Prep Kit (Illumina, CA) as per the manufacturer's instructions. The unique barcode sequences were incorporated in the adaptors for multiplexed high-throughput sequencing. The final product was assessed for its size distribution and concentration using BioAnalyzer High Sensitivity DNA Kit (Agilent Technologies, CA). The libraries were pooled and diluted to 2 nM in EB buffer (Qiagen, MD) and then denatured using the Illumina protocol. The denatured libraries were diluted to 10 pM by pre-chilled hybridization buffer and loaded onto HiSeq SR Rapid v2 flow cells on an Illumina HiSeq 2500 (Illumina, San Diego, CA) and run for 64 cycles using a single-read recipe (HiSeq Rapid SBS Kit v2, Illumina) according to the manufacturer's instructions. Illumina CASAVA pipeline (released version 1.8, Illumina) was used to obtain de-multiplexed sequencing reads (fastq files) passed the default purify filter. Reads were mapped to the mm10 transcriptome with STAR [92] and gene-level quantification performed with RSEM [93]. Differential tests were performed in DESeq2 using the SARTools pipeline [94]

The list of differentially upregulated and downregulated genes with FDR < 0.05 was imported to Ingenuity Pathway Analysis software (Qiagen, Hilden, Germany) for pathway enrichment analysis using Ingenuity Knowledge Base (IKB) as the reference set. All analysis was done using the software contextual analysis settings for mouse CD8 T cells. The enrichment significance by *P*-value between the gene list and the canonical pathway analysis was measured by Fisher's exact test. The enrichment factor is the ratio of the number of genes in a given pathway divided by the total number of genes in the pathway.

### Statistical analysis

Experimental data were analyzed on Prism 6.07 (GraphPad, La Jolla, CA) using Mann-Whitney test, one-way ANOVA, and two-way ANOVA with Tukey or Sidak’s multiple comparisons test. Error bars indicate mean ± SD. All experiments were replicated independently.

## Supporting information

S1 Fig Characterization of helped and unhelped virus-specific CD8 T cells. **(A)** Frequency of Ki-67^+^ D^b^LT359 tetramer^+^ CD8 T cells from brains (left) and spleens (right) at days 8 and 30 p.i. **(B)** gMFI of Bcl-2 on D^b^LT359 tetramer^+^ CD8 T cells from brains (left) and spleens (right). **(C, D)** Frequency of KLRG1^lo^ CD127^hi^ (left) or KLRG1^hi^ CD127^lo^ (right) D^b^LT359 tetramer^+^ CD8 T cells from brains (C) and spleens (D). **(E-H)** gMFI of T-bet (E), eomes (F), blimp-1 (G), and Tcf1 (H) in brain (left) and spleen (right) D^b^LT359 tetramer^+^ CD8 T cells at days 8 and 30 p.i. Mean ± SD of 7–12 mice per group from two-three independent experiments (A-F) and of 3–4 mice from one independent experiment (G,H). ***P<0.001, two-way ANOVA with Sidak’s multiple comparisons test (A-H).
(TIF)

S2 Fig Unhelped splenic MuPyV-specific CD8 T cells have reduced function. **(A)** Number (left) and frequency (right) of IFN-γ^+^ CD44^hi^ CD8 T cells from spleens at days 8 and 30 p.i. following ex vivo stimulation with LT359 peptide. **(B)** Quantitative PCR analysis of viral genome copies from spleen at days 8 and 30 p.i. (A & B) Mean ± SD of 6–10 mice per group from two independent experiments. *P<0.05, two-way ANOVA with Sidak’s multiple comparisons test.
(TIF)

S3 Fig bT_RM_ development is impaired in MHCII^-/-^ mice and unhelped CD8 T cells have increased expression of inhibitory receptors. **(A)** Frequency of CD103^+^ D^b^LT359 tetramer^+^ CD8 T cells from brain. **(B)** Number (left) and frequency (right) of FoxP3^+^CD25^+^ CD4 T cells at days 7 and 11 p.i. **(C,D)** TGF-β (C) and IL-21 (D) mRNA from CD4 T cells isolated from brain and stimulated with PMA/ionomycin. **(E)** Coexpression of Tim-3 and 2B4 on PD-1^hi^ D^b^LT359 tetramer^+^ CD8 T cells at days 30 (top) and 8 (bottom) p.i. **(F)** gMFI of Tim-3 and 2B4 on brain D^b^LT359 tetramer^+^ CD8 T cells at days 8 and 30 p.i. Mean ± SD of 6–8 mice per group from two independent experiments (A, E, F) or 3–4 mice from one experiment (B-D). *P<0.05, ***P<0.001, one-way ANOVA (A-D), unpaired Student’s t-test with Welch’s correction (E-F).
(TIF)

S4 Fig IgG-treated and CD4 T cell-depleted mice had similarly reduced VSV gRNA in the brain. **(A)** Quantitative PCR analysis of VSV gRNA from brain at day 4 (control) or day 35 after i.n. infection. Box and whiskers plot representing median and 5–95 percentile distribution of 4–8 mice per group from two independent experiments. **P<0.01, one-way ANOVA.
(TIF)

S5 Fig CD4 T cell depletion does not change BBB permeability, adhesion molecule expression on CD8 T cells, or extravascular location of brain CD8 T cells. **(A)** BBB permeability was measured 10 days p.i. by the accumulation of sodium fluorescein dye in the brain. **(B)** The ability of CD8 T cell depleting rat mAb given at day 10 p.i. to access spleen and brain CD8 T cells in CD4 T cell-depleted and rat IgG control-treated mice was analyzed the next day by examining colocalization of rat IgG and anti-CD8 in these organs. White arrows indicate CD8 T cells and yellow arrows CD8 T cells that were stained with both CD8 and rat IgG. **(C)** gMFI of CD49d (left), CD162 (middle), and CD11a (right) on helped and unhelped D^b^LT359 tetramer^+^ cells from blood. **(D)** Ratio of CD45^+^ (intravascular)/CD45^-^ (extravascular) total CD8 T cells and D^b^LT359 tetramer^+^ CD8 T cells from brain. Mean ± SD of 3–8 mice per group from two independent experiments.
(TIF)

S6 Fig Serum from MHCII^-/-^ mice passively immunized with αVP1 neutralized MuPyV. **(A)** LT mRNA assay showing neutralization capacity of serum from WT and MHCII^-/-^ mice at 5 days after i.c. rechallenge with MuPyV. Assay controls indicate cells treated with only IgG or VP1 mAb.
(TIF)

S7 Fig FACS-sorting strategy for CD103^-^, CD103^+^ and MHCII^-/-^-CD103^-^. **(A)** Mononuclear cells harvested from brains of B6 and MHCII^-/-^ mice at day 30 after i.c. inoculation with MuPyV were stained with D^b^LT359 tetramers, CD8, CD44, and CD103. **(B)** Heat map representing the differentially expressed pathways from the Ingenuity pathway analysis between MHCII^-/-^-CD103^-^ and CD103^-^ and MHCII^-/-^-CD103^-^ and CD103^+^.
(TIF)

S1 Table Differentially expressed genes from pathways indicated by ingenuity pathway analysis. Table indicating the–log (p-value), frequency of upregulated (indicated %**↑**) transcripts, frequency of downregulated (labeled as %**↓**) transcripts, and list of transcripts differentially expressed in each pathway.
(DOCX)
