# Supplementary figures and images for: CD4 T cells control development and maintenance of brain-resident CD8 T cells during polyomavirus infection
Source: PLoS Pathog. 2018 Oct 29;14(10):e1007365. doi: 10.1371/journal.ppat.1007365 (PMC6224182; doi:10.1371/journal.ppat.1007365)

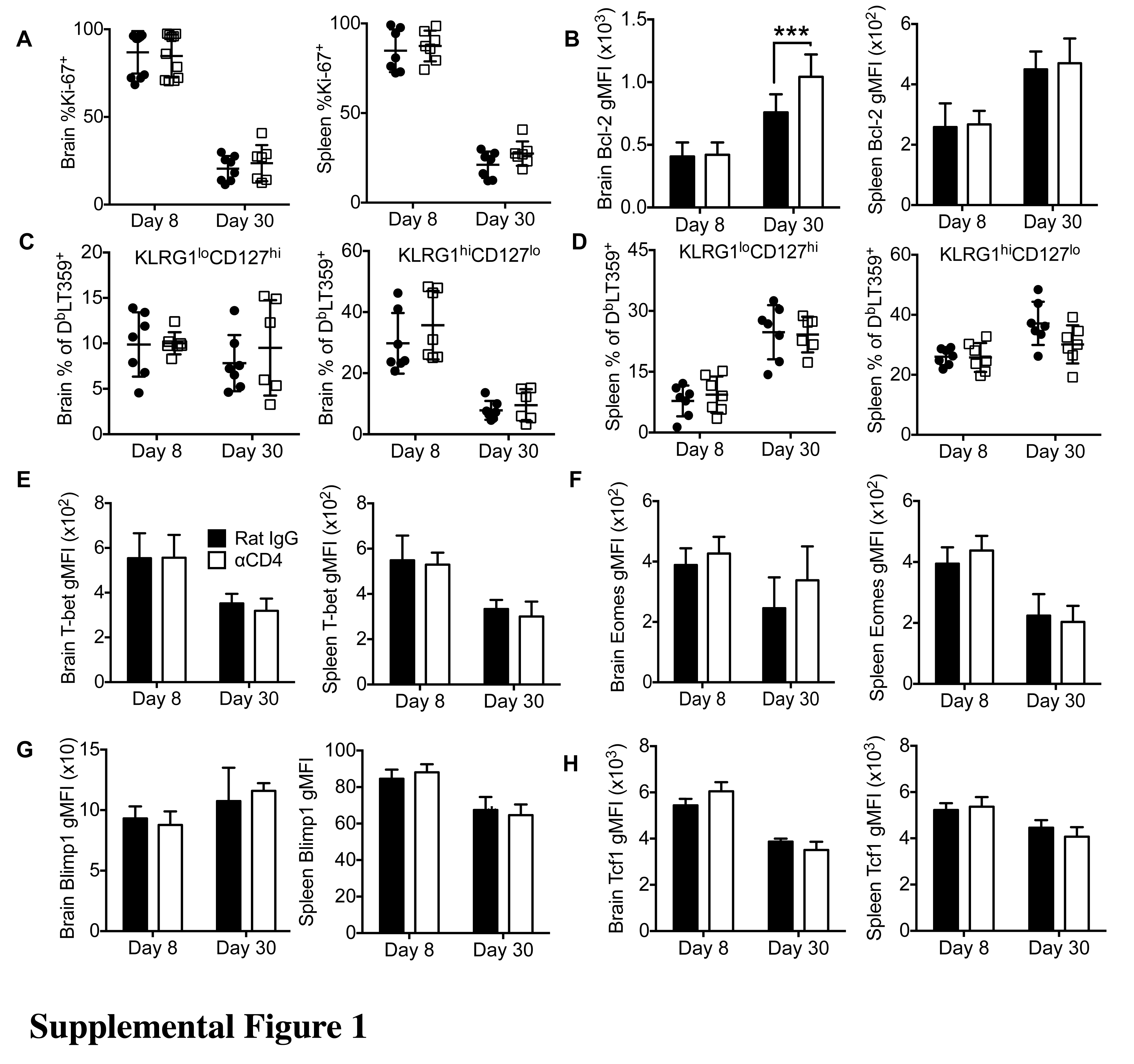

Supplement: S1 Fig — (A) Frequency of Ki-67+ DbLT359 tetramer+ CD8 T cells from brains (left) and spleens (right) at days 8 and 30 p.i. (B) gMFI of Bcl-2 on DbLT359 tetramer+ CD8 T cells from brains (left) and spleens (right). (C, D) Frequency of KLRG1lo CD127hi (left) or KLRG1hi CD127lo (right) DbLT359 tetramer+ CD8 T cells from brains (C) and spleens (D). (E-H) gMFI of T-bet (E), eomes (F), blimp-1 (G), and Tcf1 (H) in brain (left) and spleen (right) DbLT359 tetramer+ CD8 T cells at days 8 and 30 p.i. Mean ± SD of 7–12 mice per group from two-three independent experiments (A-F) and of 3–4 mice from one independent experiment (G,H). ***P<0.001, two-way ANOVA with Sidak’s multiple comparisons test (A-H). (TIF) [file ppat.1007365.s001.tif]

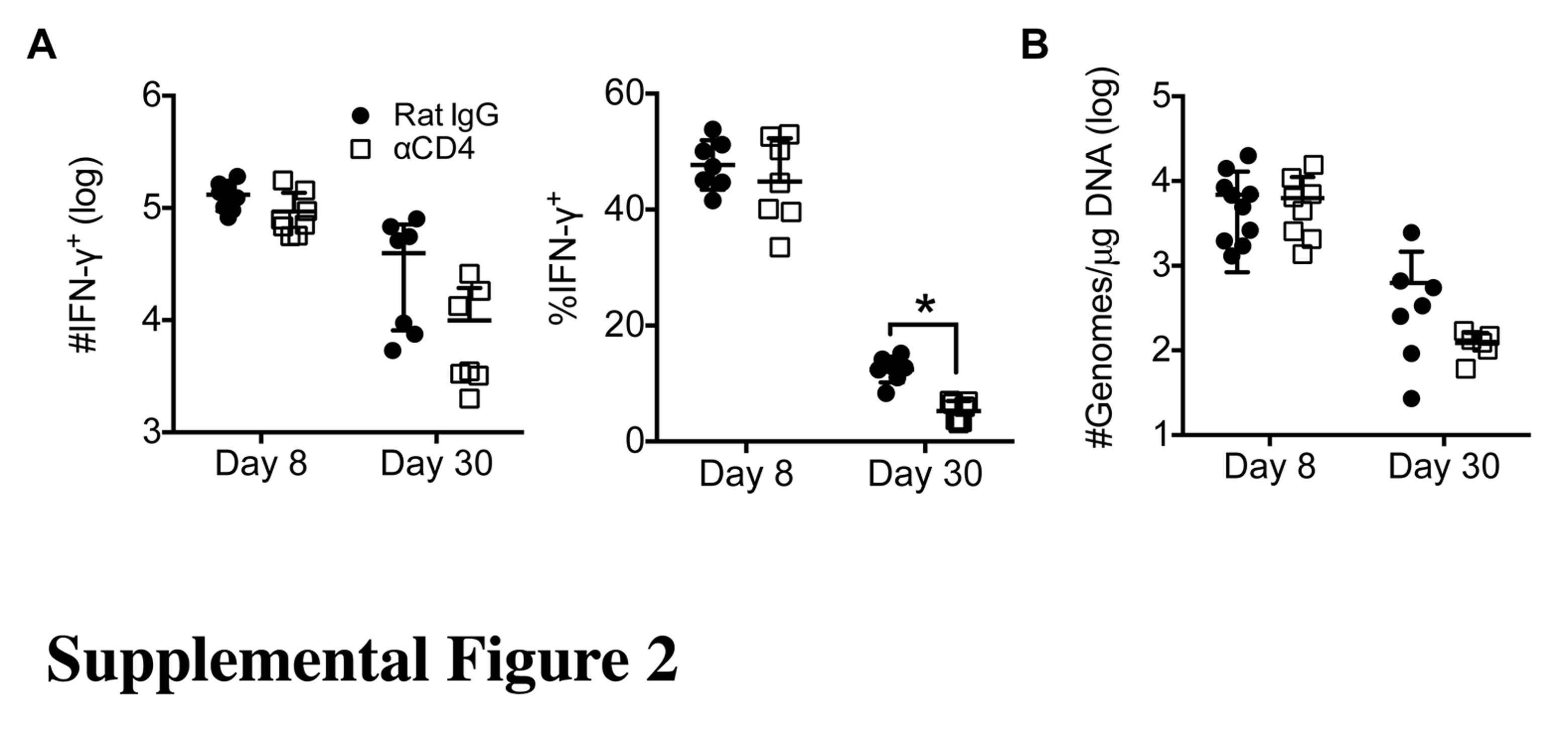

Supplement: S2 Fig — (A) Number (left) and frequency (right) of IFN-γ+ CD44hi CD8 T cells from spleens at days 8 and 30 p.i. following ex vivo stimulation with LT359 peptide. (B) Quantitative PCR analysis of viral genome copies from spleen at days 8 and 30 p.i. (A & B) Mean ± SD of 6–10 mice per group from two independent experiments. *P<0.05, two-way ANOVA with Sidak’s multiple comparisons test. (TIF) [file ppat.1007365.s002.tif]

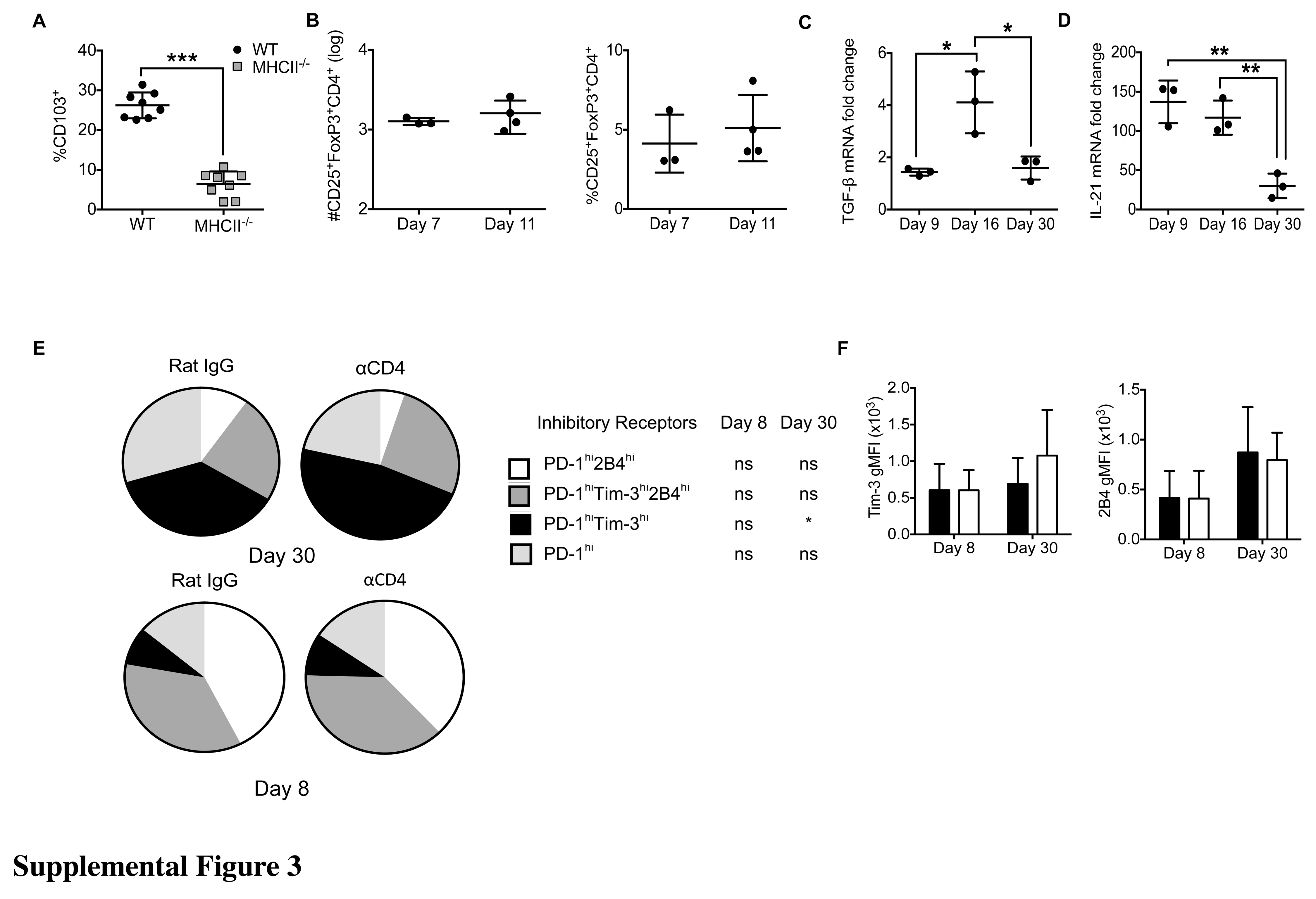

Supplement: S3 Fig — (A) Frequency of CD103+ DbLT359 tetramer+ CD8 T cells from brain. (B) Number (left) and frequency (right) of FoxP3+CD25+ CD4 T cells at days 7 and 11 p.i. (C,D) TGF-β (C) and IL-21 (D) mRNA from CD4 T cells isolated from brain and stimulated with PMA/ionomycin. (E) Coexpression of Tim-3 and 2B4 on PD-1hi DbLT359 tetramer+ CD8 T cells at days 30 (top) and 8 (bottom) p.i. (F) gMFI of Tim-3 and 2B4 on brain DbLT359 tetramer+ CD8 T cells at days 8 and 30 p.i. Mean ± SD of 6–8 mice per group from two independent experiments (A, E, F) or 3–4 mice from one experiment (B-D). *P<0.05, ***P<0.001, one-way ANOVA (A-D), unpaired Student’s t-test with Welch’s correction (E-F). (TIF) [file ppat.1007365.s003.tif]

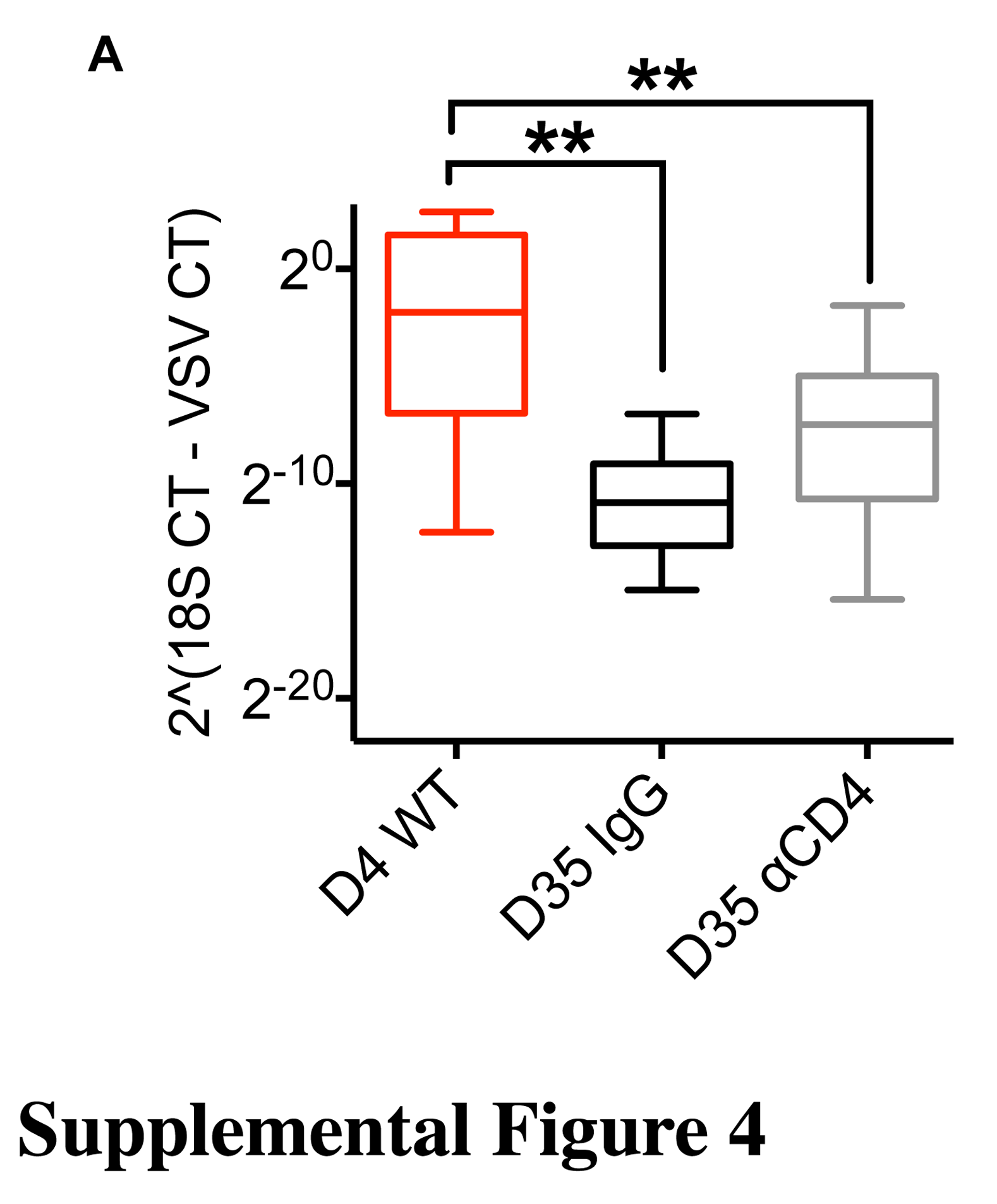

Supplement: S4 Fig — (A) Quantitative PCR analysis of VSV gRNA from brain at day 4 (control) or day 35 after i.n. infection. Box and whiskers plot representing median and 5–95 percentile distribution of 4–8 mice per group from two independent experiments. **P<0.01, one-way ANOVA. (TIF) [file ppat.1007365.s004.tif]

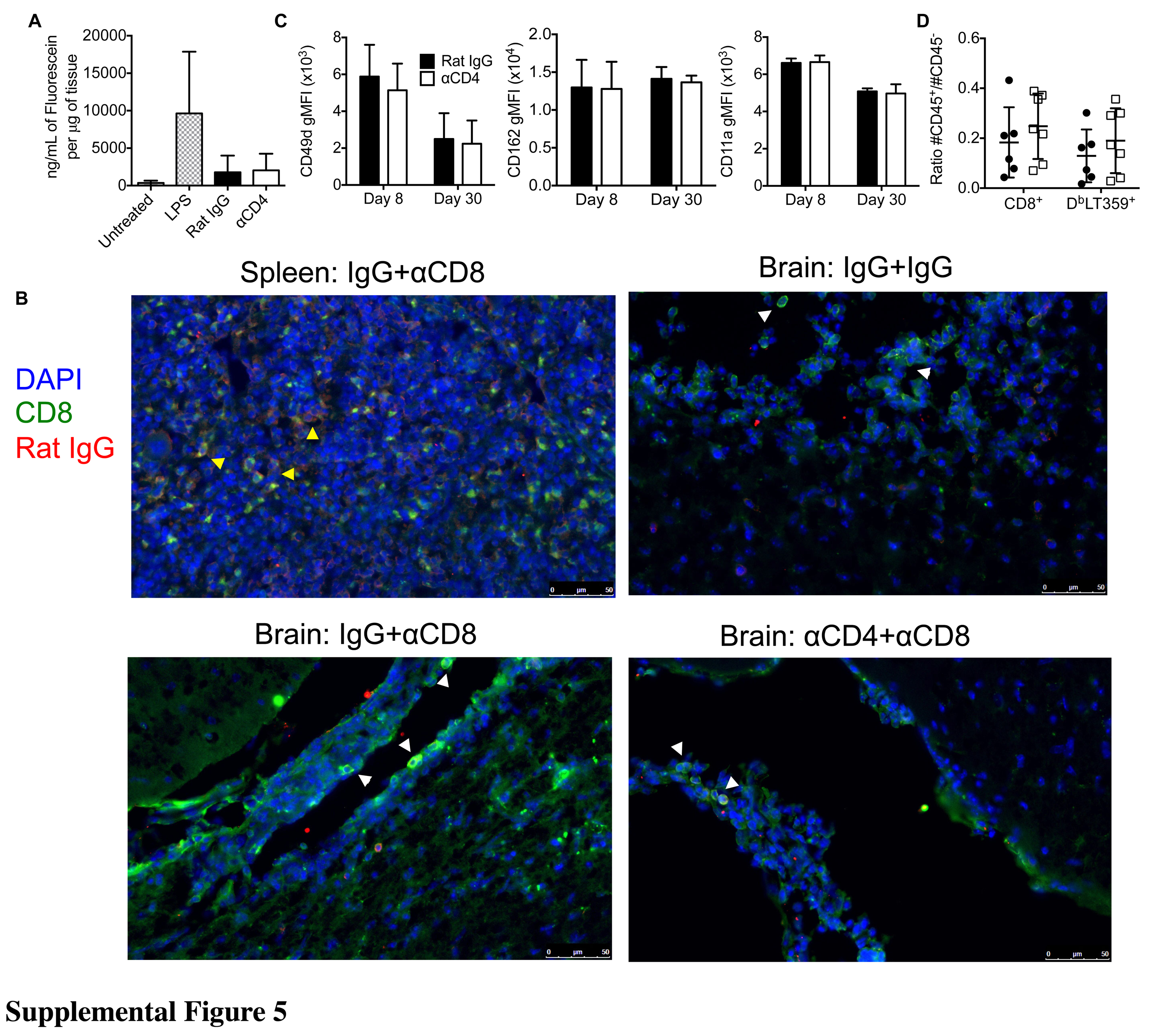

Supplement: S5 Fig — (A) BBB permeability was measured 10 days p.i. by the accumulation of sodium fluorescein dye in the brain. (B) The ability of CD8 T cell depleting rat mAb given at day 10 p.i. to access spleen and brain CD8 T cells in CD4 T cell-depleted and rat IgG control-treated mice was analyzed the next day by examining colocalization of rat IgG and anti-CD8 in these organs. White arrows indicate CD8 T cells and yellow arrows CD8 T cells that were stained with both CD8 and rat IgG. (C) gMFI of CD49d (left), CD162 (middle), and CD11a (right) on helped and unhelped DbLT359 tetramer+ cells from blood. (D) Ratio of CD45+ (intravascular)/CD45- (extravascular) total CD8 T cells and DbLT359 tetramer+ CD8 T cells from brain. Mean ± SD of 3–8 mice per group from two independent experiments. (TIF) [file ppat.1007365.s005.tif]

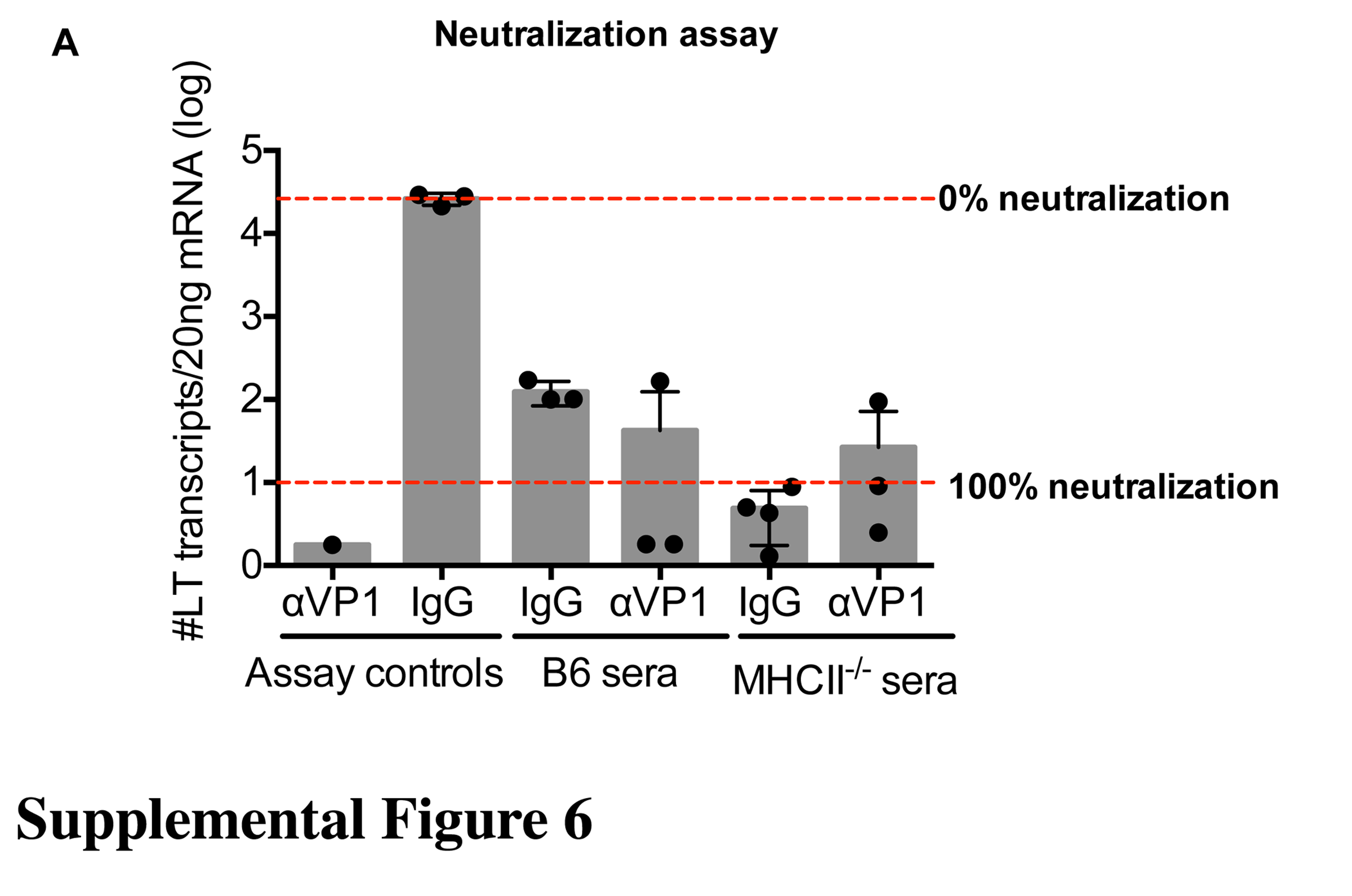

Supplement: S6 Fig — (A) LT mRNA assay showing neutralization capacity of serum from WT and MHCII-/- mice at 5 days after i.c. rechallenge with MuPyV. Assay controls indicate cells treated with only IgG or VP1 mAb. (TIF) [file ppat.1007365.s006.tif]

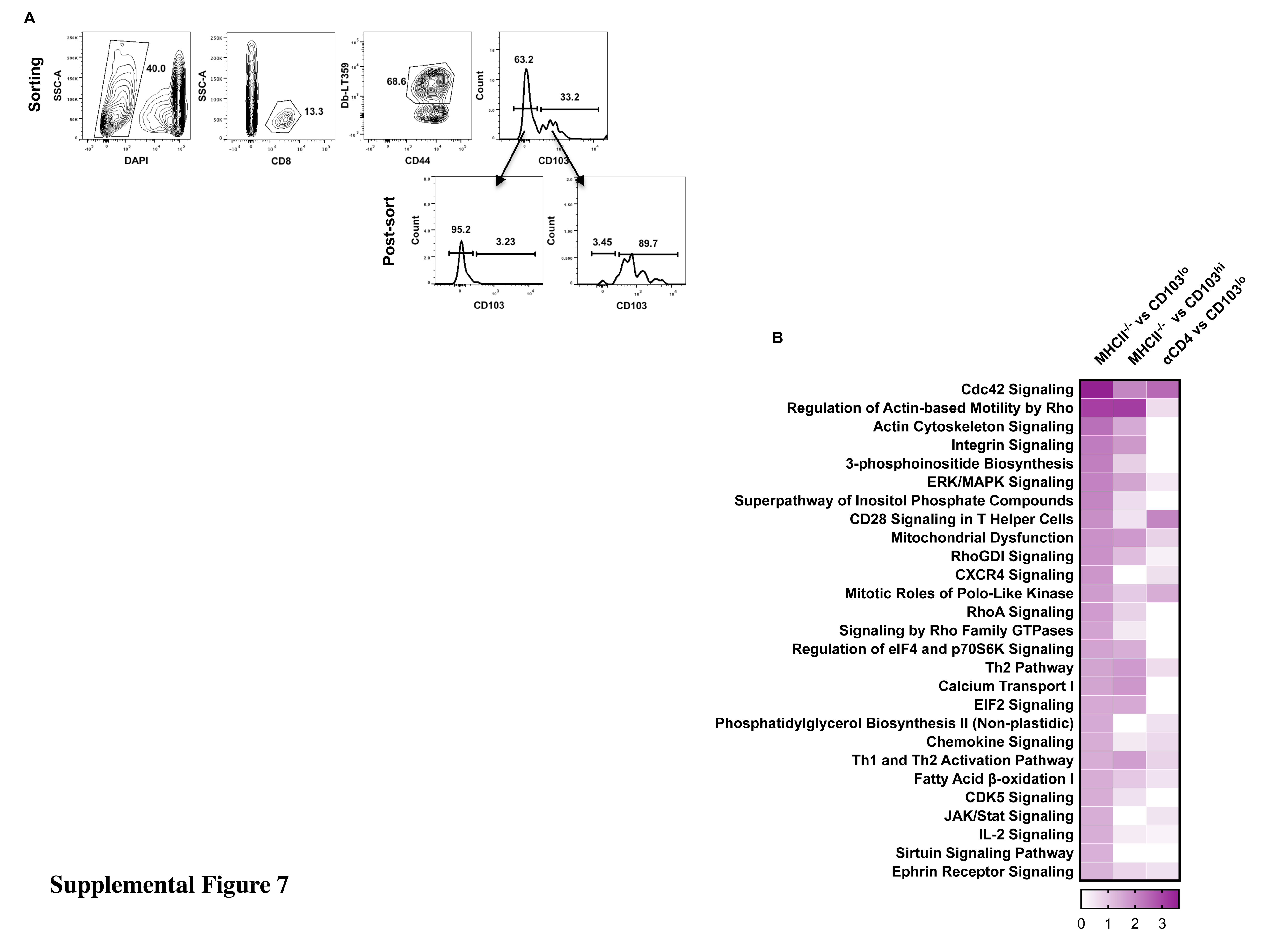

Supplement: S7 Fig — (A) Mononuclear cells harvested from brains of B6 and MHCII-/- mice at day 30 after i.c. inoculation with MuPyV were stained with DbLT359 tetramers, CD8, CD44, and CD103. (B) Heat map representing the differentially expressed pathways from the Ingenuity pathway analysis between MHCII-/--CD103- and CD103- and MHCII-/--CD103- and CD103+. (TIF) [file ppat.1007365.s007.tif]
