# Supplementary material for: CD4 T cells control development and maintenance of brain-resident CD8 T cells during polyomavirus infection
Source: PLoS Pathog. 2018 Oct 29;14(10):e1007365. doi: 10.1371/journal.ppat.1007365 (PMC6224182; doi:10.1371/journal.ppat.1007365)
Supplement: S1 Table — Table indicating the–log (p-value), frequency of upregulated (indicated %↑) transcripts, frequency of downregulated (labeled as %↓) transcripts, and list of transcripts differentially expressed in each pathway. (DOCX) [file ppat.1007365.s008.docx]

**Table S1:** **Differentially expressed genes from significantly enriched pathways by Ingenuity pathway analysis**

| Pathway name | -log(p-value) | %🡹 | %🡻 | Gene List |
| --- | --- | --- | --- | --- |
| Cdc42 signaling | 3.62 | 6 | 7 | CD3G, FOS, JUN, PPP1R12A, ARPC2, PAK2, EXOC2, HLA-DOB, ARPC3, MYL12B, MYL12A |
| Regulation of Actin motility by Rho | 3.07 | 2 | 12 | RAC2, PFN1, PPP1R12A, ARPC2, PAK2, ARPC3, MYL12B, MYL12A |
| Actin Cytoskeleton Signaling | 2.38 | 3 | 6 | RAC2, PFN1, PPP1R12A, GNA12, ARPC2, PAK2, TRIO, ARPC3, TLN1, MAP2K1, MYL12B, MYL12A |
| Integrin Signaling | 2.20 | 2 | 6 | RAC2, CAPNS1, PFN1, PPP1R12A, ARPC2, PAK2, ITGA6, ARPC3, TLN1, MAP2K1, MYL12B, MYL12A |
| 3-phosphoinositide Biosynthesis | 2.15 | 6 | 2 | PTPN6, PPP1R12C, SYNJ1, PTPN2, PPP1R12A, DUSP1, PPP1R13B, ICOS, MTMR2, PPP2R5E, DUSP2 |
| ERK/MAPK signaling | 2.09 | 4 | 4 | RAC2, FOS, PPP1R12A, PPP2CA, DUSP1, PAK2, TLN1, PPP2R5E, MAP2K1, DUSP2, EIF4EBP1 |
| Superpathway of inositol phosphate compounds | 2.07 | 6 | 2 | PTPN6, PPP1R12C, SYNJ1, PTPN2, PPP1R12A, DUSP1, PPP1R13B, ICOS, MTMR2, PPP2R5E, DUSP2, PPIP5K2 |
| CD28 signaling in T helper cells | 1.91 | 6 | 3 | CD3G, FOS, PTPN6, JUN, ARPC2, HLA-DOB, ARPC3, MALT1, MAP2K1 |
| Mitochondrial Dysfunction | 1.88 | 1 | 7 | HSD17B10, SDHB, NDUFS8, NDUFB7, ATPAF1, NDUFB8, GPX4, AIFM1, APP, PINK1 |
| RhoGDI Signaling | 1.86 | 3 | 5 | PPP1R12C, PPP1R12A, GNA12, ARPC2, PAK2, ARPC3, ARHGDIB, MYL12B, MYL12A |
| CXCR4 Signaling | 1.79 | 5 | 3 | FOS, JUN, EGR1, GNA12, PAK2, LYN, MAP2K1, MYL12B, MYL12A |
| Mitotic Roles of Polo-like Kinase | 1.70 | 4 | 7 | HSP90AB1, PPP2CA, PLK3, PPP2R5E, RAD21 |
| RhoA Signaling | 1.68 | 2 | 6 | PFN1, PPP1R12A, GNA12, ARPC2, ARPC3, MYL12B, MYL12A |
| Signaling by Rho Family GTPases | 1.61 | 4 | 3 | FOS, PPP1R12C, JUN, PPP1R12A, GNA12, ARPC2, PAK2, ARPC3, MAP2K1, MYL12B, MYL12A |
| Regulation of eIF4 and p70S6K Signaling | 1.58 | 4 | 3 | PPP2CA, RPS23, RPS21, EIF4A2, RPS15A, EIF2S3, PPP2R5E, MAP2K1, EIF4EBP1 |
| Th2 Pathway | 1.56 | 3 | 5 | RUNX3, CD3G, IL2RG, JUN, MAF, ICOS, CXCR6, HLA-DOB |
| Calcium Transport I | 1.54 | 0 | 25 | ANXA5, ATP2B4 |
| EIF2 Signaling | 1.49 | 5 | 2 | RPL22, RPL18A, RPS23, RPL39, RPL26, RPS21, EIF4A2, RPS15A, EIF2S3, MAP2K1, RPLP0 |
| Phosphatidylglycerol Biosynthesis II (Non-plastidic) | 1.47 | 10 | 5 | AGPAT5, GPAT4, MBOAT7 |
| Chemokine Signaling | 1.43 | 7 | 2 | FOS, JUN, PPP1R12A, CCL5, MAP2K1 |
| Th1 and Th2 Activation Pathway | 1.43 | 3 | 4 | RUNX3, CD3G, IL2RG, JUN, MAF, IL6R, ICOS, CXCR6, HLA-DOB |
| Fatty Acid β^2^-oxidation | 1.42 | 0 | 14 | HSD17B10, ECHS1, SCP2 |
| CDK5 Signaling | 1.41 | 5 | 3 | PPP1R12A, PPP2CA, EGR1, ITGA6, PPP2R5E, MAP2K1 |
| JAK/STAT Signaling | 1.39 | 7 | 1 | FOS, PTPN6, JUN, PIAS1, SOCS4, MAP2K1 |
| IL-2 Signaling | 1.37 | 5 | 4 | FOS, IL2RG, JUN, CSNK2B, MAP2K1 |
| Sirtuin Signaling Pathway | 1.36 | 1 | 4 | TIMM8A, POLR2F, SDHB, GADD45B, JUN, NDUFS8, NDUFB7, NDUFB8, H1F0, LDHA, ABCA1, APP |
| Ephrin Receptor Signaling | 1.32 | 2 | 5 | RAC2, RGS3, GNA12, ARPC2, PAK2, SH2D3C, ARPC3, MAP2K1 |
